# Global abundance estimates for 9,700 bird species

**DOI:** 10.1073/pnas.2023170118

**Published:** 2021-05-17

**Authors:** Corey T. Callaghan, Shinichi Nakagawa, William K. Cornwell

**Affiliations:** ^a^Centre for Ecosystem Science, School of Biological, Earth and Environmental Sciences, UNSW Sydney, Sydney, NSW 2052, Australia;; ^b^Ecology & Evolution Research Centre, School of Biological, Earth and Environmental Sciences, UNSW Sydney, Sydney, NSW 2052, Australia

**Keywords:** global biodiversity, abundance, rarity, SADs, data integration

## Abstract

Quantifying the abundance of species is essential to ecology, evolution, and conservation. The distribution of species abundances is fundamental to numerous longstanding questions in ecology, yet the empirical pattern at the global scale remains unresolved, with a few species’ abundance well known but most poorly characterized. In large part because of heterogeneous data, few methods exist that can scale up to all species across the globe. Here, we integrate data from a suite of well-studied species with a global dataset of bird occurrences throughout the world—for 9,700 species (∼92% of all extant species)—and use missing data theory to estimate species-specific abundances with associated uncertainty. We find strong evidence that the distribution of species abundances is log left skewed: there are many rare species and comparatively few common species. By aggregating the species-level estimates, we find that there are ∼50 billion individual birds in the world at present. The global-scale abundance estimates that we provide will allow for a line of inquiry into the structure of abundance across biogeographic realms and feeding guilds as well as the consequences of life history (e.g., body size, range size) on population dynamics. Importantly, our method is repeatable and scalable: as data quantity and quality increase, our accuracy in tracking temporal changes in global biodiversity will increase. Moreover, we provide the methodological blueprint for quantifying species-specific abundance, along with uncertainty, for any organism in the world.

## Significance

For the fields of ecology, evolutionary biology, and conservation, abundance estimates of organisms are essential. Quantifying abundance, however, is difficult and time consuming. Using a data integration approach integrating expert-derived abundance estimates and global citizen science data, we estimate the global population of 9,700 bird species (∼92% of all extant bird species). We conclude that there are many rare species, highlighting the need to continue to refine global population estimates for all taxa and the role that global citizen science data can play in this effort.

Abundance (i.e., the number of individuals of a species) is a fundamental component of ecology, evolutionary biology, and conservation (1–13). For example, knowledge of abundance provides insights into the evolutionary mechanisms underlying intra- and interspecific population dynamics (14), the structure of communities and metacommunities across space and time (15), and the relative commonness and rarity of species within a community necessary for conservation prioritization (16, 17). Abundance of a species is structured by many ecological processes, and there is debate about which are the key processes in a simple but sufficient ecological model (8). An improved estimation of species abundance distributions (SADs) has been and will continue to be the most important empirical piece of evidence in this debate (18–21). Yet, despite the importance of SADs in ecology, evolution, and conservation, small spatial scale data has been all that exists to inform our empirical understanding of SADs (22–24), thus limiting the generality of our understanding of abundance. Recently, Enquist et al. (12) proposed that SADs should be extended to the global scale (i.e., global SAD; hereafter gSAD) to elucidate the general patterns of abundance beyond the idiosyncrasies of small spatial-scale studies. Such global-scale abundance data will improve our understanding of the following: fundamental macroecology questions such as the structure of abundance across biogeographic realms or across feeding guilds (3, 25); important biogeography questions such as the relationship between range size and abundance (26, 27); important evolutionary questions such as the relationship between body size and population abundance (4, 5, 28, 29); and many other emerging questions in eco-evolutionary dynamics (30, 31). Therefore, to address this knowledge gap, we derived a repeatable and scalable methodology, relying on data integration, to provide species-specific global abundance estimates for nearly all the world’s bird species (92%) and consequently a gSAD focused on absolute abundances.

**Fig. 1.**
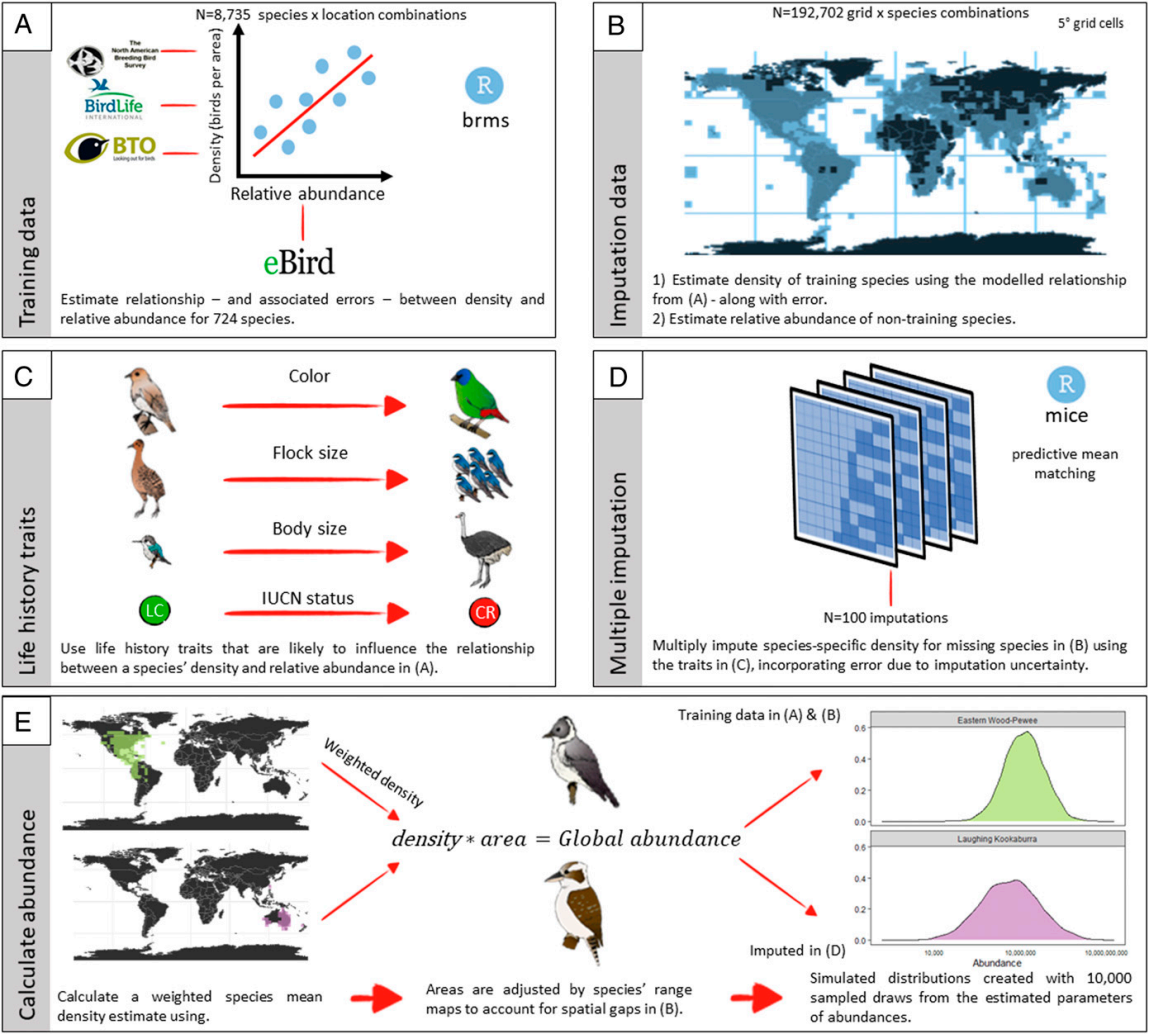
A methodological overview of our statistical approach to estimate species-specific abundances. (*A*) First, we modeled the relationship between relative abundance in eBird and the “true” density of a species in a given region. (*B*) We then collated data throughout the world, calculating relative abundance of each species in 5° grid cells. (*C*) We collated life history traits which were likely to influence the relationship between a species’ density and relative abundance. (*D*) We performed multiple imputation to impute density for missing species in each 5° grid cell throughout the world. (*E*) We calculated a weighted density for each species in which predicted density in every grid cell was weighted by the number of checklists in those corresponding grid cells. This helped to incorporate the heterogeneous distribution of densities throughout the world. We then adjusted these density estimates using a species’ range map to simulate an abundance distribution which incorporated measurement error and uncertainty.

Global-scale data sources of abundance are heterogeneous, often with few species’ global abundances estimated. Creating a systematic global data collection effort to estimate abundance for a given taxa (e.g., through distance sampling) is logistically prohibitive (32). Additionally, the few studies which model abundance at regional or continental scales (12, 33) are generally limited in taxonomic coverage (i.e., failing to fully sample all potential species in the regional or continental pool of species). One of the most successful approaches to providing data at broad spatial (e.g., global) scales is data integration, in which small sets of high-quality data are used to inform much larger but less precise data (34). This general approach has progressed the entire field of remote sensing, in which, for example, high-quality on-the-ground data informs remote spectral measurements (35). We apply this same general data integration framework to solve previous shortcomings of abundance estimation by integrating expert-derived population estimates of bird abundance with global citizen science data (36). This approach allows us to estimate species-specific abundance for 9,700 species of bird—about 92% of all extant bird species. First, we modeled the relationship between relative abundance (i.e., average abundance per effort) from eBird citizen science data and density (i.e., total individuals per unit area) from a suite of expert-derived population estimates for 724 bird species. We then collated ecological and life history traits (i.e., body size, color, threat status, and flock size) that are likely related to the detectability of a species for the majority of the 9,700 species in our dataset and used the densities for the training species to perform multiple imputation— predicting each species’ density while accounting for both uncertainty and imputation error (Fig. 1 and *Methods*). Based on a weighted density, accounting for geographic sampling biases, and the global area a species encompasses, each species in our analysis received a simulated distribution of possible abundances to account for uncertainty in our modeling process (Fig. 1). These species-specific abundance distributions (Fig. 1*E*) can then be statistically aggregated to calculate the number of individual birds at any taxonomic (e.g., species, genus, family, order, and class) or ecological (e.g., biogeographic realms, feeding guilds) grouping.

**Fig. 2.**
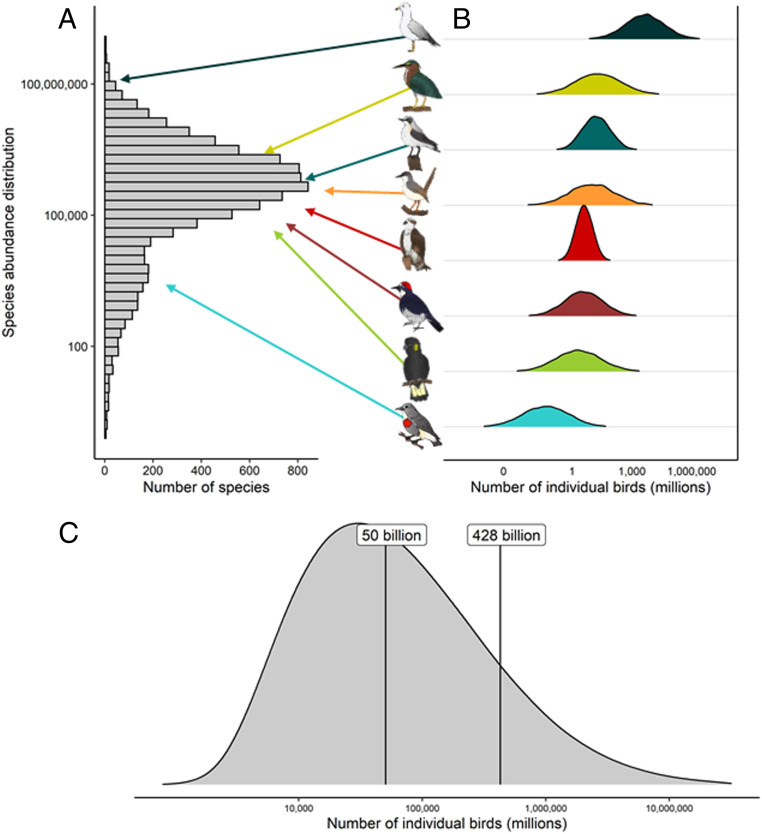
(*A*) The gSAD, calculated using the median of each species’ simulated abundance distribution and adding a constant 1 for those species predicted to have 0 abundance. (*B*) Examples of species’ simulated abundance distributions. Species shown from top to bottom are: Ring-billed Gull; Green Heron; Northern Wheatear; Ashy Prinia; Osprey; Acorn Woodpecker; Yellow-tailed Black-Cockatoo; and Midget Flowerpecker. (*C*) The total distribution of the number of individual birds in the world, calculated by summing all species-specific abundance distributions for 9,700 bird species (e.g., those from *B*). The average of all 9,700 global population estimates was 5.2 million, whereas the median was 450,000.

We calculate that there are likely to be ∼50 billion individual birds in the world at present: about six birds for every human on the planet. This represents the midpoint of our estimates (i.e., the median), albeit with considerable uncertainty (Fig. 2). Compared with the median estimate, the mean estimate of the aggregated distribution for all birds in the world was ∼428 billion individual birds (Fig. 2). While we provide an estimate with a wide highest-density interval, our estimate corresponds well with a previous estimate of the number of individual birds in the world by Gaston and Blackburn (37), who estimated that there were between 200 and 400 billion individual birds in the world. Notably, Gaston and Blackburn (37) did not estimate species separately but rather extrapolated from small-scale density estimates in which all bird species were considered equal. We, however, provide data for nearly all the world’s bird species.

We constructed a gSAD by treating the median of the species-specific simulated abundance distributions as that species’ global population estimate (Fig. 2). The global abundance estimates for the 9,700 species considered in our analysis clearly show a log left–skewed distribution (Fig. 2*A*). The skewness of this gSAD was −0.972 (95% CI: −1.028, −0.914). We show that there are very few abundant species and many rare species at the global scale (7, 8, 24, 38, 39). While we acknowledge that we did not encompass every extant species in our analysis, there are three main instances leading to a species not being included: 1) the species is exceedingly rare and has not been sampled by eBird; 2) the species is sampled in a region of the world with very few eBird sampling events, leading to potentially unreliable relative abundance measures (*Methods*); and 3) the species is sampled by eBird but marked as “sensitive,” meaning these data are not publicly available. In all three instances, the species excluded from our analysis are directly (i.e., not sampled) or indirectly (i.e., marked as sensitive due to, for example, threats from the bird trade) rare in nature. Therefore, we highlight that our gSAD may be conservative, and the remaining ∼8% of unsampled species here probably fall along the tail of the gSAD representing rare species.

Many species in our analysis have population estimates that are very small: 1,180 species (12%) have population estimates of <5,000 individual birds; about 200 more species than expected if the gSAD followed a truly log-normal distribution. Conversely, relatively few species are very abundant. The top 10 most abundant birds in the world, and their approximate global population estimates, are House Sparrow (1.6 billion), European Starling (1.3 billion), Ring-billed Gull (1.2 billion), Barn Swallow (1.1 billion), Glaucous Gull (949 million), Alder Flycatcher (896 million), Black-legged Kittiwake (815 million), Horned Lark (771 million), Sooty Tern (711 million), and Savannah Sparrow (599 million). The estimated abundance and associated uncertainty of all 9,700 species in our analysis can be found in Dataset S1.

While it is clear that there is a predominance of rarity at a global scale, the mechanisms generating this gSAD—and local SADs—remain largely unknown. Understanding the heritability, or nonheritability, of abundance can provide insights into how abundance distributions are generated. If rarity is heritable at the species level, then extinction risk would be unequally spread across the bird phylogeny, with extinction threat highly stratified across different clades (40). Thus far, the pursuit of this question at local and regional scales has resulted in inconclusive results: some have found that abundance and/or rarity is more similar among closely related species (41–43), whereas others have not (44–46). However, different study systems can lead to idiosyncratic results (47), potentially an artifact of spatial scale (48). Whether rarity is phylogenetically conserved at a global scale remains untested, and this is important because it can provide insights into the evolutionary mechanisms generating the gSAD while quantifying the phylogenetic structure of extinction risk. We assessed the heritability of abundance at a global level by 1) testing for phylogenetic signal, which implies species-level heritability (49), and 2) assessing the hierarchical distribution of rarity (i.e., the generality of the log left–skewness of the gSAD) by using a taxonomically nested analysis and calculating the skew of the global abundance distribution at species, genus, family, and order levels (sensu ref. 50).

**Fig. 3.**
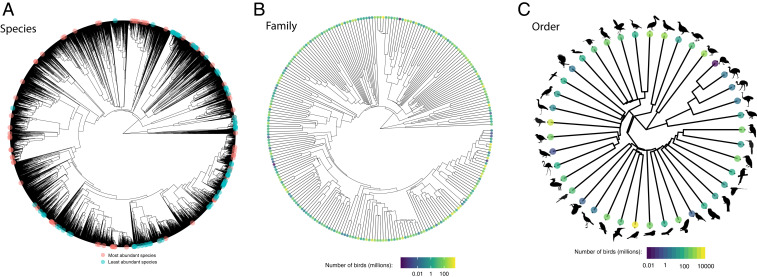
Phylogenetic representation at the (*A*) species, (*B*) family, and (*C*) order level showing the global abundance of individual birds in the world.

Our analysis showed that commonness and rarity at the species level are spread throughout the tips of the phylogenetic tree, leading to an overall lack of phylogenetic signal (Blomberg’s K = 0.014 ± 0.109 [SE], *P* = 0.45; considering phylogenetic uncertainty, refer to *Methods*), albeit with clusters in some clades (Fig. 3*A*). We found strong evidence that the abundance distribution follows a log left–skewed distribution across taxonomic levels and that there was a decline in the magnitude of skewness from species to order (*SI Appendix*, Fig. S1). To test the robustness of this pattern, we performed a resampling analysis and found that the mean values of skewness were (*SI Appendix*, Fig. S2): −0.89 (95% CI: −0.88, −0.91) at the species level; −0.86 (95% CI: −0.87, −0.84) at the genus level; −0.83 (95% CI: −0.85, −0.82) at the family level; and −0.80 (95% CI: −0.81, −0.79) at the order level (an alternative bootstrapping approach found similar results to these; *SI Appendix*, Fig. S3). This decline in magnitude of the proportion of rarity at the tips from species to order, combined with the lack of phylogenetic signal, suggests an important role for recent speciation in creating global abundance patterns. Visual clusters of abundant species are apparent from the phylogeny (Fig. 3*A*), but these clades also contain rare species (*SI Appendix*, Fig. S4), which is consistent with the weakening of the log left-skew pattern at higher taxonomic levels. Local studies may find that closely related species are characterized by similar levels of abundance (41–43, 48), but our results show that this pattern weakens decisively at the global scale. This suggests that abundance cannot be directly inherited through a speciation event, although traits that drive abundance (i.e., range size, body size, and habitat breadth) may persist through the event. Our results show that direct nonheritability of abundance predominates at the global scale with implications for the phylogenetic distribution of extinction risk. While most work surrounding rarity focuses on three axes—local abundance, geographic range size, and habitat breadth—our work highlights the need to consider also the global abundance. Future research should focus on extending this empirical work to other taxa and across spatial scales (8, 12) to better understand the mechanisms leading to SADs and gSADs, and the implications of global rarity.

**Fig. 4.**
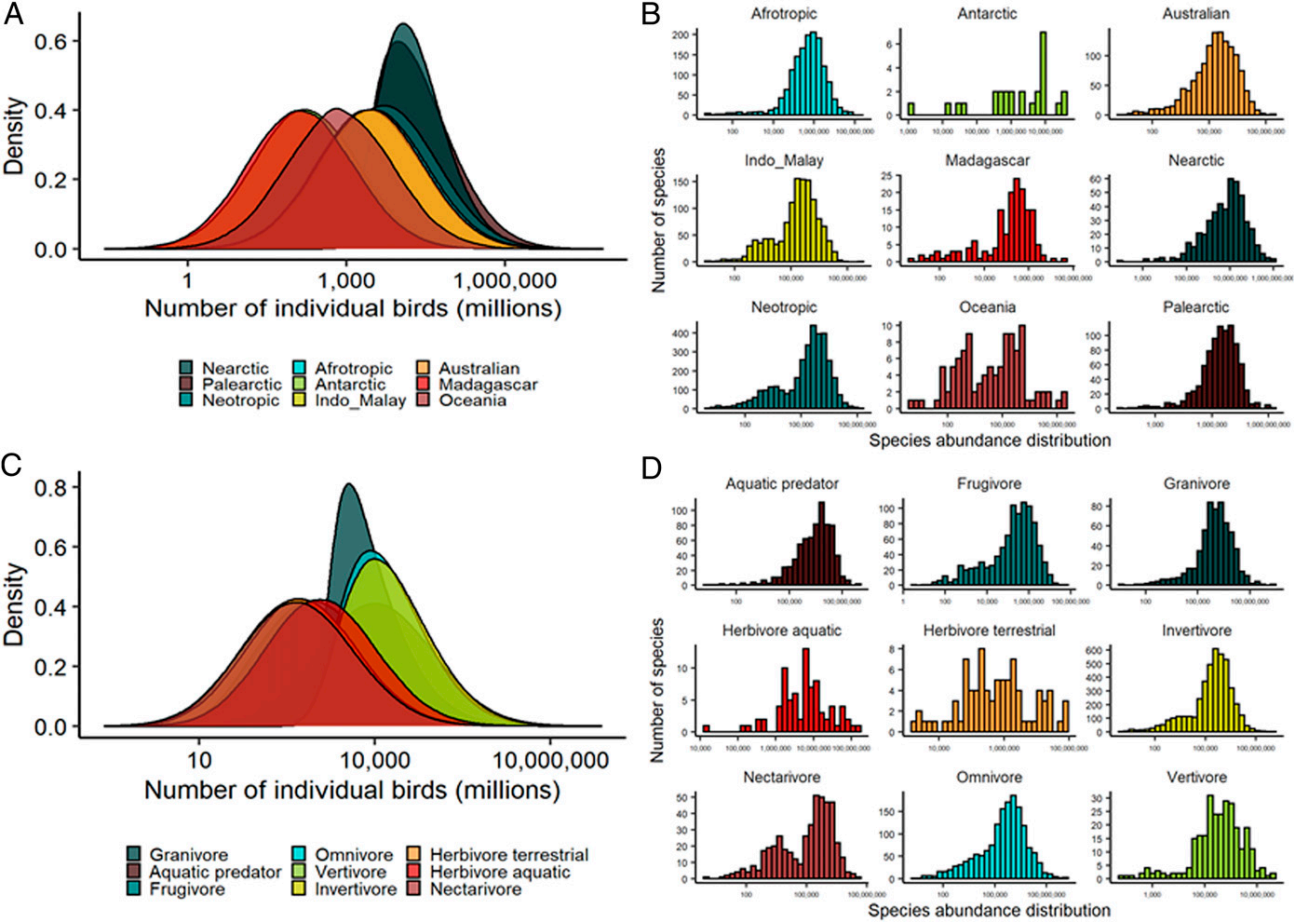
(*A*) The distribution of the number of individual birds, calculated by summing all species-specific abundance distributions (e.g., Fig. 2*B*) categorized within specific biogeographic realms (N = 9,178 species). (*B*) The SAD for each biogeographic realm, in which each species’ median abundance estimate is used. (*C*) The distribution of the number of individual birds, calculated by summing all species-specific abundance distributions (e.g., Fig. 2*B*) categorized within specific feeding guilds (N = 9,157 species). (*D*) The SAD for each feeding guild, in which each species’ median abundance estimate is shown. Species’ classifications were taken from ref. 54. The feeding guild of scavenger is not shown because very few species were assigned as scavenger.

We can also use the species-specific global population estimates, as opposed to summing the species-specific abundance distributions (*SI Appendix*, Fig. S5), to assess the abundance distribution of species within a given biogeographic realm or feeding guild. We again found strong support for log left–skewed abundance distributions both within biogeographic realms (skewness = −1.50, −0.06; Fig. 4*B*) and feeding guilds (skewness = −1.46, −0.03; Fig. 4*D*). The empirical evidence that rare species are indeed more common than simple models predict suggests that conventional theory is not sufficient, and additional mechanisms need to be considered (8).

In the face of ongoing biodiversity loss (51), there is an urgent need for conservation prioritization. Such prioritization can be improved by moving past species-specific planning to also incorporate conservation of higher-order taxonomic clades that are both phylogenetically unique (52, 53) and have overall low global abundance. Our approach allows for these data to be easily quantified, providing global estimates per taxonomic clade (e.g., Fig. 3). We found that the least abundant orders of birds in the world (Fig. 3*C*) were kiwis (3,000) and mesites (154,000), contrasting with the most abundant orders of birds which were perching birds (28 billion), shorebirds (9.7 billion), and waterfowl (2.3 billion). The same procedure can be carried out for families (e.g., Fig. 3*B*) or even genera. Similarly, conservation prioritization can focus on biogeographic realms (i.e., protecting the most important habitats to conserve biodiversity) or functional diversity (i.e., prioritizing conservation of species in the least abundant feeding guilds). In this light, we also estimated the number of individual birds in both biogeographic realms and feeding guilds (following classification by ref. 54) by aggregating the abundance distributions based on a species’ biogeographic realm and feeding guild categorization and taking the median of these aggregated distributions. We find that the majority of the world’s individual birds are from the palearctic (18 billion) and nearctic (16 billion) biogeographic realms (Fig. 4*A*), whereas there are far fewer birds in the Madagascar (1.3 billion) and Antarctic (1.6 billion) biogeographic realms. Among feeding guilds (Fig. 4*C*), invertivores (15 billion) and omnivores (13 billion) are the most abundant groups of birds in the world, contrasting with scavengers (194 million) and nectarivores (479 million). We currently provide the necessary data (Dataset S1) to understand the current populations of birds at large scales, helping current conservation efforts for birds. However, importantly, our data integration approach is easily repeatable, providing a means to potentially track temporal changes in global biodiversity at a myriad of different taxonomic or ecological classifications.

One key feature of our analysis is that it provides error estimates—we propagate error throughout the analysis (cf. ref. 37). As data are heterogeneously distributed, this will necessarily lead to some species being better characterized than others. It is likely difficult to appropriately estimate abundance of exceedingly rare species because of two possible instances: 1) it is possible that citizen scientists will preferentially observe the rarest species, potentially inflating their citizen science–generated relative abundance within a given region and thereby leading to an overestimation of their global population, or 2) species may be so exceedingly rare that they have too little data to make informed population estimates because they are only observed a handful of times. Our approach may be less certain for specific clades, based on life history. As an example, seabirds are colonial nesters, often breeding on remote islands in immense flocks, rarely encountered during this phase of their annual cycle by birders, and are therefore more likely encountered in small flock sizes during nonbreeding periods of their annual cycle, which could influence their relative abundance calculations. Conversely, shorebirds are unlikely to be encountered during their breeding season when they breed throughout the remote tundra but most likely to be encountered by citizen scientists when they form large congregations during the nonbreeding phase of their annual cycle. We accounted for some of these biases by taking monthly means of relative abundance and averaging across temporal and spatial biases to generate a single mean density estimate across space and time (*Methods*). Currently, our approach is limited by the training data used in our analyses, and increasing the number of training species will likely improve the certainty of abundance estimate for a number of species (Fig. 1*E* and *Methods*). As a consequence, some species have relatively narrow ranges of their abundance estimates compared with others (Fig. 2; Dataset S1). Unsurprisingly, the training data in our analysis was strongly biased toward countries with a historic commitment to bird monitoring (e.g., United States of America and the United Kingdom), and this is well illustrated by the narrower range of distributions for Nearctic and Palearctic birds in our analysis compared with other biogeographic realms (e.g., Fig. 4*A*).

Although we currently provide only a static “snapshot” of global population abundances, it is important to note that our approach of integrating fine-scale abundance estimates with massive-scale citizen science data will continue to grow in its strength and validity. As citizen science continues to increase in quality and quantity (55, 56), so too will the validity of our approach. Concomitantly, as the global push for open-data principles in biodiversity conservation continues to increase (57), the necessary training data (i.e., localized expert-derived abundance estimates) to improve our statistical approach will likely become increasingly available. Moreover, although we currently focus on birds in the present manuscript, the data integration approach can act as a blueprint for quantifying species-specific abundance, along with uncertainty, for any organism in the world. Future research, then, should focus on three key goals: 1) increasing the certainty surrounding species-specific abundance estimates, 2) developing automated pipelines which will allow our approach to be easily repeated (i.e., updated annually or biannually), providing a method to track temporal change in global biodiversity abundance at different spatial scales, and 3) developing generalized approaches to measure abundance for other taxa. We are confident that all three of these goals are achievable in the near term.

In the more immediate term, we illustrate how our results will prove useful to further address a suite of fundamental and longstanding questions across the ecological and evolutionary subdisciplines. What are the population dynamics of species in space and time? How is a species’ global abundance related to its life history (e.g., *SI Appendix*, Fig. S6)? How is abundance influenced by anthropogenic habitat changes? Which species, genera, families, or orders are most worthy of future conservation attention (e.g., Fig. 3)? All of these questions can start to be answered with spatial and taxonomic coverage that has never before been possible. There are a considerable number of individual birds in the world—∼50 billion—but fully understanding why and how they all arrived at their current population sizes will be paramount to the future study of evolution, ecology, and conservation.

## Methods

Our approach to estimate species-specific global abundances for 9,700 species can be broken down into five key steps, outlined in turn below (Fig. 1):

• Step 1 (Training data): Model the relationship between known (i.e., externally validated best available data) density estimates and relative abundance from eBird to derive a species-specific training model, while incorporating known error in the relative abundance estimates.
• Step 2 (Imputation data): Calculate a measure of relative abundance for all species in 5° grid cells throughout the world. For the training species, calculate density using the results from Step 1 in each unique grid a species occupies.
• Step 3 (Life history traits): Collate life history traits (bird color, flock size, body size, and International Union for Conservation of Nature [IUCN] status) that are likely to influence the relationship between the true population of a species and the relative abundance of a species calculated through eBird.
• Step 4 (Multiple imputation): Perform multiple imputation by chained equations to predict the density of a species and its uncertainty per grid cell, based on the known relationship between estimated density and observed relative abundance for our training species and the traits collated at Step 3.
• Step 5 (Calculate abundance): Use the predicted densities and uncertainties to derive a mean global density estimate for each species and multiply this density estimate by the observed area of a species—with extrapolation where necessary and possible—to calculate a simulated global abundance distribution.

In the following methods, we expand on each of these key steps.

### Training Data.

#### Abundance estimates.

Our main objective was to quantify density of bird species throughout the world. Fundamental to density is the measure of absolute abundance—the known, or estimated, quantity of individuals in a population. Estimating the total population size of a given animal population is a fundamental research question in ecology and conservation (58). Much research has investigated how to best estimate absolute abundances, with many techniques having been applied to estimate abundances (59, 60). When abundance is known for a region, then the density is simply:

$$Density=\;\frac{Abundance}{Area}.$$

Thus, for our analysis, it was critical to find external population estimates that had an estimated population abundance for a given geographic region. Because most reporting schemes derive from a government initiative to understand which species are most at risk (61), most population estimates are based on geopolitical boundaries, and total species-specific populations are then scaled up based on range size extrapolations. Our analysis thus relied, to some extent, on populations within geopolitical boundaries. We used published abundance estimates from three sources: 1) the Partners in Flight Population Estimates Database (62); 2) population estimates from the British Trust for Ornithology (63); and 3) from BirdLife International Data Zone datazone.birdlife.org/home. We collated a total of 724 species for which we had estimated population abundance. Each training species’ abundances were calculated in either geopolitical boundaries (i.e., for species extracted from the Partners in Flight database and the British Trust for Ornithology) or throughout their entire geographic range (i.e., for species extracted from the BirdLife Data Zone). For example, estimates from the Partners in Flight database were available stratified to each state and Bird Conservation Region throughout the United States where that species was found. Each of these data sources are treated in more detail in *SI Appendix*, *SI Methods*.

#### Relative abundance estimates.

We extracted relative abundance estimates from eBird (36, 64) citizen science data. Here, we defined the relative abundance to mean the number of birds observed per some unit effort (e.g., time and/or distance). eBird was launched in 2002 by the Cornell Lab of Ornithology and currently has >800 million global bird observations. Volunteer birdwatchers submit “checklists” of birds seen and/or heard while birdwatching. Species, or counts of species, which are unexpected based on the spatiotemporal coordinates of the observations, are flagged and reviewed by an extensive network of expert volunteers before being accepted into the dataset (65). Each checklist is marked as either “complete” or “incomplete” by the volunteer birdwatchers submitting the data. This distinction indicates whether they are submitting a complete list of all birds seen and/or heard during their observation period. We only used complete checklists in our analysis as this allows for absences (i.e., nondetections) to be inferred. For our analysis, we used the eBird basic dataset (version ebd_relMay2019). We aggregated eBird data from January 2010 to May 2019. We acknowledge that some species may experience changes in their population sizes during this time, but we note that 10 y is the IUCN-recommended duration to calculate population change when generation time is not known (66). To further ensure these data represent the “best quality” data, we employed an additional set of filtering, aiming to remove potential “outliers” which could bias our dataset (67–70). We only included complete checklists, checklists >5 min and <240 min in duration, and checklists which traveled <5 km. However, some potential mistakes are still possible in the eBird dataset (see an example below).

To date, eBird data have been used for a variety of abundance-related measures. The general approach is to measure “relative abundance”: the number of birds counted when accounting for time spent birdwatching and distance traveled while birdwatching. For example, the Cornell Lab of Ornithology currently models relative abundance in space and time for >800 of the most common species in North America and elsewhere: https://ebird.org/science/status-and-trends/. Because our general approach was based on geopolitical boundaries, and there would be vastly different numbers of available data among different geopolitical boundaries (cf. USA and a remote Indonesian island), we aimed for a simple and tractable modeling approach that would generalize to anywhere that eBird data are collected. As such, after initial exploration (*SI Appendix*, Fig. S7 and *SI Methods*), we used the mean abundance across all checklists (including checklists when a species was not identified; zeros) as our measure of relative abundance.

#### Modeling the relationship between density and relative abundance.

Using the known abundance estimates from external sources described above, we calculated the density per each geopolitical region or species’ range (*SI Appendix*, Fig. S8), corresponding to the relative abundance measure from eBird. Both relative abundance and density were log10 transformed, and any values that were initially zero (i.e., not detected in eBird but present in the external data sources) were set to −4.5 (log10 scale) given the minimum value was −4.499787 in the dataset (*SI Appendix*, Fig. S9). We quantitatively checked the sensitivity of including these zeros on the overall effect of our model and found that the random intercepts and slopes for the species that were kept were robust when some observations were removed, and therefore, we chose to include the zeros in our model fitting process, as described above. We were left with a total of 8,735 data points of 724 species in which most species had only one observation in the model, but some species had comparatively many observations used in the model (*SI Appendix*, Figs. S10 and S11). We then fit a Bayesian mixed-effects random slope model using the R package brms (71, 72), which is a wrapper to fit Bayesian models in stan (73) via rstan (74). This model is equivalent to a Type II regression model, in which we explicitly modeled the error in our eBird relative abundance measures. The error of the eBird relative abundance measures was calculated as the SD of all mean estimates (i.e., SE) for a given species’ time (i.e., month) times space (i.e., geopolitical region) measures of mean abundance, with a small sample size correction, followed by the delta method to convert this to log10 scale. Although error estimates are available for most of our training datapoints (i.e., the density estimate; see above), this is not available for all data points. Therefore, we decided not to include this measurement error on our response variable. This approach is 1) inclusive by allowing for more species to be included in the modeling procedure by not omitting species without error for the training data and 2) conservative by propagating a larger amount of SE surrounding the intercept and slope (i.e., uncertainty) forward in our modeling framework. We used log10 density as the response variable and log10 relative abundance as the fixed effect, with species as random intercepts and log10 relative abundance as corresponding random slopes. We used 10,000 iterations and four chains, with a warmup of 2,000. We used the default priors from brms which are weakly informative, having only minimal influence on the estimations, while improving convergence and sampling efficiency. In the case of the Gaussian distribution, sigma has a half student *t* prior that scales in the same way as the group-level SDs (71, 72). From this brms model, we extracted the random slope and intercept for each species (*SI Appendix*, Fig. S12), which provided a two-parameter equation (*y* = *mx* + *b*) that signified the relationship between the observed density of a species and relative abundance from eBird (*SI Appendix*, Figs. S9–S12). In addition, we extracted the SE (i.e., the SDs of the posterior distributions) of the intercept and the slope for each species in the training dataset (*SI Appendix*, Fig. S12). It is essential to carry forward these errors for random intercepts and slopes as each species would have a differing amount of error associated with its intercept and slope (75).

##### Brms model validation.

We used a leave-one-out approximation (76, 77) from the brms package to check the diagnostics of our brms model and found that 95% of observations had a Pareto k < 0.7—in the “ok” range (71, 76, 77), suggesting that very few datapoints could be considered “influential” in our model fitting process. A Bayesian approximate R^2^—calculated as the variance of the predicted values divided by the variance of predicted values plus the expected variance of the errors (78)—for this model was 0.78. To further validate the brms model used to extract species-specific intercepts and slopes, we used the extracted intercept and slope for each species with the original observed data (see *Methods*, above) to test whether the brms model could accurately predict the external estimates of total population abundance. We found that the intercepts and slopes extracted from the brms strongly predicted abundance estimates for our training species (*SI Appendix*, Figs. S13 and S14) with an R^2^ of 0.88. Ultimately, we found that our brms model was robust to extract species-specific estimates of intercept, slope, and the SE of the intercept and slope—see below for overall workflow validation demonstrating the robustness of this model further.

### Imputation Data.

After we had modeled the relationship between observed density and relative abundance, we were left with a two-parameter model (*y* = *mx* + *b*) describing this statistical relationship that helps to account for the noise in relative abundance measures. We then derived a 5 × 5° spatial grid covering the world (*SI Appendix*, Fig. S15). We only used grids with a minimum of 50 eBird checklists within at least one month (*SI Appendix*, Fig. S16). Within each grid (N = 579), we calculated the relative abundance of each species as defined above: a mean abundance across all checklists, including zeros for checklists on which a species was not found. This was stratified by month. If a species was not observed in a grid (i.e., a relative abundance of 0), then we assumed that the species does not exist in that grid. Using our two-parameter model, which included SEs for both the intercept and slope, we assumed that the correlation between slope and intercept would be −1 (*SI Appendix*, Fig. S13); this is because an overestimated intercept (higher intercepts) will almost always result in shallower slopes, creating the intercept–slope correlation of −1. Under this assumption, we calculated the density—and its SE—for 684 of our training species (i.e., the ones that were found after criteria to filter grids were employed and limited the overall number of species to be included in further analyses) within each grid that training species was observed (*SI Appendix*, Fig. S17). After collapsing the variability among months within each grid to a single value by averaging the relative abundances, we were left with a total of 192,702 species × grid combinations. A total of 41,652 of these had density estimates across a total of 684 species.

While the eBird project has strong and stringent review protocols (36, 64) and an extensive network of regional volunteers (65), some errors and mistakes can still be made. If a species was available in the eBird dataset version that we used, we did not do any “cleaning” of known (or presumed) mistakes. For example, we predicted a positive abundance of the largely recognized-as-extinct Ivory-billed Woodpecker because there were positive observations of this species in the eBird version we used (see above). Therefore, our results presented in Dataset S1 should be interpreted carefully based on known biology of a species and prior expectations (main text). However, eBird—and other citizen science datasets—is continually growing in both quantity and quality, and these mistakes or errors are continuously being rectified in updated versions of the dataset.

### Life History Traits.

Our main objective through multiple imputation was to impute the missing density estimates by modeling the relationship between estimated density derived from the training data and the relative abundance for the nontraining species. However, the relationship between observed density and relative abundance from eBird checklists (e.g., Fig. 1*A*) is likely to be influenced by a suite of species’ life history traits. Previous work has shown that species traits (e.g., body size, color, and group size) can influence the detectability of a species (79–84), and thus in turn, the likelihood a species is recorded in a citizen science dataset (85). Sólymos et al. (81) and Johnston et al. (82) found that body size was a significant predictor of bird species’ detectability. There is also support that group size can influence detectability of animals (83). Although difficult to quantify, it is thought that the coloration of an organism influences its detectability (84). Lastly, the overall abundance class (e.g., common versus rare) of a species will also likely influence a species’ detectability and likelihood of being recorded in the eBird citizen science dataset (85). Based on the above biological understanding in detectability, in our imputation, we included auxiliary data on the following: species’ color, flock size, body size, and IUCN status. For color, we used a dataset of >5,000 species (86) and calculated two separate metrics: brightness and distance from brown. For flock size, we used eBird to calculate the overall mean flock size among all presences in the dataset for each species. For body size, we used the adult body mass (in grams) as extracted from ref. 87. Lastly, we used the IUCN status for species in the imputation (88) as an ordinal variable, extracted from the BirdLife International working list of birds version 3, available here: datazone.birdlife.org/species/taxonomy.

Prior to imputation, we indeed found that these traits corresponded reasonably well with different components (i.e., either the intercept or slope or SE of the intercept or slope) of the detectability of species between observed density and eBird relative abundance (*SI Appendix*, Figs. S18 and S19). A species’ intercept showed strong correlation with body mass (*r* = −0.467) and moderate correlation with color (*r* = 0.101 and *r* = 0.145 for distance from brown and brightness, respectively). Whereas flock size was weakly correlated with intercept (*r* = −0.086) and moderately correlated with slope (*r* = 0.146), it was strongly correlated with the SE of the intercept (*r* = −0.626) and slope (*r* = −0.524). A similar pattern as flock size was shown for the IUCN status as an ordinal variable. All pairwise correlations between intercept, slope, SE of the intercept, SE of the slope, and the species’ traits can be seen in *SI Appendix*, Fig. S18. After we calculated density for the training species, using the intercepts and slopes, there remained moderate to strong relationships between the relative abundance, the estimated density, and the various life history traits (*SI Appendix*, Fig. S19), indicating that the life history traits are likely to moderate the relationship between relative abundance and density.

### Multiple Imputation.

Our “imputation” dataset included 192,702 rows (grid × species) with 11 variables: 1) species ID (0% missing), 2) the log-transformed number of eBird checklists (0% missing), 3) the number of months a species was observed in that grid (0% missing), 4) relative abundance as described in detail above (0% missing), 5) flock size (0% missing), 6) IUCN status (8% missing), 7) body size (16% missing), 8) color distance from brown (40% missing), 9) color brightness (40% missing), 10) estimated density (79% missing), and 11) the SE for the density (79% missing). These 11 variables showed moderate to strong correlations (*SI Appendix*, Figs. S18 and S19). Our targets for imputation were density (at the specific grid level) and its SE (10 and 11), mainly informed from relative abundance (0% missing values) but also helped by the five auxiliary variables (5 through 9). These auxiliary variables had good coverage; for example, 79% of the rows (153,095) had at least one of these auxiliary variables present. Among the variables with missing values, the first five (5 through 9) were species-level variables while the last (10 and 11) were at the observation (row)-level. We used the R packages mice (89) and miceadds (90) to conduct two-level multiple imputation in which mixed models with one clustering (i.e., random) factor were used to impute missing data using a predictive mean matching algorithm (2lonly.pmm for the species-level variables and 2l.pmm for the observation-level variables). In our case, the clustering (i.e., random) factor was the species. We created 100 imputed datasets.

#### Multiple imputation model validation.

To validate our multiple imputation modeling approach, we performed three different checks, recommended to assess the reliability and plausibility of multiple imputation (91, 92). First, we performed a qualitative assessment of external checking and found no biologically unreasonable imputed estimates (92). Second, we compared the density of observations for the training data and the density of observations for the imputed data. Using 10 randomly chosen imputations—of the 100 total imputations we performed in our analysis—we visually inspected the imputed density and density SE estimates compared with the observed density and density SE estimates (*SI Appendix*, Fig. S20). We found that all 10 of our randomly chosen imputations matched closely with the observed densities used during the imputation, suggesting that the imputed values are statistically plausible. Third, we performed a leave-one-out cross-validation analysis, in which, because our imputation was nested at the species level (i.e., species was treated as a random effect), we left each of our training species out, one at a time (e.g., *SI Appendix*, Fig. S21). For this analysis, each imputation modeling process was repeated as described above but with only 10 imputations, as opposed to the 100 imputations we used in our full model, for computational reasons. We found that at the observation level, the range of the 10 imputations encompassed the observed density estimate for 95% of leave-one-out imputed observations (e.g., *SI Appendix*, Fig. S21). Similarly, the range between the 0.05 and 0.95 quantiles of our 10 imputations encompassed the observed density for 92% of observations. When assessing the data at the observation level, the predictive power of our imputation method to impute density was very strong: a linear mixed-effects model with the response variable as imputed density and the predictor variable as observed density with a random effect for species had a marginal R^2^ of 0.84. When taking the mean density estimates among grids for each species, we similarly had strong predictive power and a linear model had an R^2^ of 0.48 (*SI Appendix*, Fig. S22). Overall, our multiple imputation produced reliable density estimates, and we also note that we accounted for the SE of the imputation procedure throughout further analyses (see below).

### Calculate Abundance.

Because some species can occur very rarely in a grid (i.e., one time), representing an out-of-range observation, for instance, we weighted the grid densities by the number of checklists a species occurs on in a grid divided by the number of total checklists in that grid. This provides a single density for every species that represents the density which is weighted from the grids where that species is most frequently observed, and thus, the density is likely to be most reliable (*SI Appendix*, Fig. S23). More generally, collapsing species’ densities among grids to a mean density helps to account for the known differences in density throughout a species’ range (93) by taking the average density estimate, incorporating the low-density and high-density regions of a species’ range. Across our 9,700 species, the average number of grids a species occupied was 19, with a SD of 36 (*SI Appendix*, Fig. S17). We note that many species are only found in one grid cell, and although their densities may differ throughout their range, we were unable to account for this potential bias, but the differences in densities are likely to be greatest for species with larger ranges (93). We also calculated imputed SEs for the density as the square root of the sum of: 1) imputation variance of the density and 2) the square of the average of the imputed SEs, weighted by the number of checklists. Because some large, and biologically unreasonable, SEs were observed initially, we set a ridge prior for the SE of the density of 1 for predicted density, which equates to 91 times higher density than the point estimate of the density in terms of the upper confidence limit. We consider this prior to be conservative and likely biasing our estimates, leading to overly large confidence intervals in some instances. Accordingly, each species received a mean density estimate (*SI Appendix*, Fig. S24) and a SE of that density (*SI Appendix*, Fig. S25) in a given grid cell.

For each species in our dataset, we calculated a mean density estimate (on the log10 scale) of all grid cells a species was found in, as described above. We then calculated the total area occupied by each species in our analysis by summing the area of the number of grid cells each species was found in. However, we adjusted these areas in two directions, depending on a species’ range size. First, for species with relatively small ranges that can be smaller than the average grid cell size (24,000 square miles), we clipped the area of that species to its known range size (*SI Appendix*, Fig. S26 shows such an example). Second, because eBird data are not homogeneous throughout the world (e.g., Fig. 1*B*), there are many gaps in our sampled grid cells (e.g., Siberia and Africa—*SI Appendix*, Fig. S15). Therefore, for a species whose estimated range was not fully sampled by our eBird analysis (i.e., the grid cells used did not fully cover that species’ range), we used the area of the total range size—as opposed to the total area of grids a species was found in—with the observed mean density estimate from our analysis. Estimated species’ ranges were provided by BirdLife International. However, we note that we did not have data for every species’ range in our analysis given taxonomic differences between Clements and BirdLife International taxonomy and a general lack of data for some species. Therefore, our range adjustment was not done for 2,731 species, in which case we assumed that the total area of their sampled grid cells approximates the total species range size. Whether a species’ abundance estimate was adjusted based on range is noted in Dataset S1.

Using the mean density estimate (on the log10 scale) and a species’ range size, we then obtained estimates of abundance, drawing 10,000 random draws from a normal distribution with the mean density estimate and the corresponding SE. We defined the median of this distribution (Fig. 1*E*) as the species-specific global abundance. We also report on the lower and upper 95% CIs in Dataset S1.

This method was repeated for every species in our analysis (N = 9,700). To determine the overall number of individual birds in the world, we summed the species-specific distributions as described above, ensuring the distributions were ordered before summing (i.e., the smallest values or largest values are summed as we combined distributions). By ordering the distributions before summing, we ensured that the likelihood of values particular to each species corresponded with one another, therefore ensuring that the middle values corresponded to those with the highest likelihood. While this approach was done for every species, we can also sum species-specific distributions based on any classification of a species (e.g., genus, family, order, biogeographic realm, and feeding guild) as we did in our analysis.

### Overall Workflow Validation.

To validate our overall workflow to calculate species-specific abundance estimates, we performed a leave-one-out cross-validation approach for the 684 training species that were included in both the brms model and the multiple imputation step of our analysis. For each species, we removed that species from our training pool and reperformed the analysis—including the brms model, the multiple imputation model, and the simulated distribution of abundance estimates. For this process, to avoid computational bottlenecks, the brms model was specified the same but fitted with only two chains, 3,000 iterations, and a warmup of 1,000, while the multiple imputation was fitted over 10 imputations each time (qualitative checks for a handful of species showed minimal differences when more iterations and/or imputations were employed). We then were able to estimate a species-specific abundance distribution for each species when it was 1) included as a training species in the full workflow and 2) when it was entirely withheld as a training species (e.g., *SI Appendix*, Fig. S27). In most instances, the distribution of possible abundance estimates was wider (i.e., wider confidence intervals) when a species was not included in the process, compared with when it was included in the full model, and in some instances, the distributions were very similar (*SI Appendix*, Fig. S27). Importantly, we found very strong correlation between the median population abundance for each species when withheld from the analysis and the median population abundance from the full model when all species were included (*SI Appendix*, Fig. S28)—with an R^2^ of 0.94.

### Assessing the Phylogenetic Signal of Species-Level Abundance Estimates.

Using the function phylosig from the R package, phytools (94), we calculated Blomberg’s K as our measure of phylogenetic signal. To incorporate phylogenetic uncertainty, we used 250 phylogenetic trees based on ref. 95 and aggregated resulting statistics, using Rubin’s rule described in ref. 96 by assuming *P* values from randomization tests are comparable to those from *t* distributions (97).

### Assessing the Skewness of Abundance Distributions.

In addition to summing species-specific distributions of abundance and calculating the total median estimate (on the original scale) for each family, order, biogeographic realm, and feeding guild, we also calculated an abundance distribution for each of these categories—with the addition of genus—by summing the species-specific median estimates for each category. Both these approaches showed strong similarity (*SI Appendix*, Figs. S2 and S3). We used the skewness function from the package e1071 (98) to calculate the skew of a given abundance distribution. To assess the robustness of our finding that the species, genus, family, and order abundance distributions were log left skewed, we employed a resampling approach. First, we randomly sampled a quantile (from 0.1 to 0.99) and took the abundance estimate at that quantile for each species (as opposed to the median estimate which is presented throughout the main text) and then calculated the skewness of the SAD, as well as the genus, family, and order abundance distributions (calculated by summing the species-specific median estimates of abundance) for each random sample. We performed this 1,000 times to derive a distribution of skewness measures, demonstrating the robustness of our finding that abundance distributions—across taxonomic levels—are log left skewed (*SI Appendix*, Fig. S2). In addition, we performed a bootstrapping approach to bootstrap CIs of the skewness measures, corroborating our resampling approach (*SI Appendix*, Fig. S3).

## Supplementary Material

Supplementary File

Supplementary File

## Data Availability

eBird data are freely available to download from https://ebird.org/data/download. Population estimates were extracted from refs. 62 and 63 and http://datazone.birdlife.org/home. Range maps used to adjust population areas are available from BirdLife International. All trait data are freely available through the sources mentioned above. Code and necessary data to reproduce our analyses are available from Zenodo, https://doi.org/10.5281/zenodo.4723365 (99).
