# Supplementary material for: Global abundance estimates for 9,700 bird species
Source: Proc Natl Acad Sci U S A. 2021 May 17;118(21):e2023170118. doi: 10.1073/pnas.2023170118 (PMC8166167; doi:10.1073/pnas.2023170118)
Supplement: Supplementary File [file pnas.2023170118.sapp.pdf]

PNAS
www.pnas.org

Supplementary Information for

Global abundance estimates for 9,700 bird species

Corey T. Callaghan[1, 2, 3], Shinichi Nakagawa[2], and William K. Cornwell[1, 2]

[1]*Centre for Ecosystem Science; School of Biological, Earth and Environmental Sciences; UNSW Sydney; Sydney, NSW, Australia*

[2]*Ecology & Evolution Research Centre; School of Biological, Earth and Environmental Sciences; UNSW Sydney; Sydney, NSW, Australia*

[3]Corresponding author: Corey T. Callaghan
Email: c.callaghan@unsw.edu.au

This PDF file includes:

Supplementary Methods text
Figures S1 to S28
SI References

Other supplementary materials for this manuscript include the following:

Dataset S1

Supplementary Information Text

Methods.

We used published abundance estimates from three sources: (1) the Partners in Flight Population Estimates Database[1]; (2) population estimates from the British Trust for Ornithology[2]; and (3) from BirdLife International datazone http://datazone.birdlife.org/home.

Partners in flight population estimates database. We extracted data from the Partners in Flight Population Estimates Database[1], downloaded from: http://pif.birdconservancy.org/PopEstimates/. This dataset provides population estimates for breeding North American landbirds at multiple geographic scales, following the 'Partners in Flight approach'[3,4,5]. This dataset represents the best-available population estimates for North American landbirds. It predominantly relies on data from the North American Breeding Bird Surveys scheme, based on the decade 2006—2015, but also complements these data with data from other schemes; e.g., Ontario atlas point count data[6]. Although the North American Breeding Bird Surveys were not designed to specifically estimate population sizes, the data are collected in a single standardized method which involves random start points and revisiting the same points each breeding season[7] forming a fundamental component to the methodological approach[5].

In short, the 'Partners in Flight approach' takes a species count proportional to the area sampled, multiplies this value by the area of the region, and accounts for detection adjustments. In order to adjust for detection probability, the approach adjusts for: (1) time

of day, (2) detection distance, and (3) the likelihood of detecting both members of a breeding pair[3,4,5]. The approach then relies on a Monte Carlo simulation to estimate uncertainty in the population estimate for each species[5], providing a distribution of population ranges for each species. Importantly, population estimates are initially derived within geo-political regions — based on state and Bird Conservation Region overlaps — and are then used as the building block for a continental-wide population estimate. The underlying sample sizes used to derive these estimates, which are proportional to the area of the geo-political region, is reflected in the population uncertainty[5]. This approach has received some thoughtful critiques[8,9,10], and the latest version of this approach addresses these in more detail[11]. For full details on the methods used to derive the population estimates, see the Handbook to the Partners in Flight Population Estimate Database, Version 3.0[1].

Population estimates of birds in Great Britain. We extracted population estimates from Musgrove et al. 2013[2] for the geographic region of Great Britain. Specifically, we extracted data from their Appendix 1. This dataset is a collection of many different sources and estimates of population size for both breeding and wintering populations — depending on the species. Ultimately, it presents the work of millions of hours of effort by the British ornithological community[2]. For many species, they rely on the British Breeding Bird surveys and distance sampling, because the British Breeding Bird surveys collect data in different distance bands. Because Musgrove et al.[2] rely on a suite of different methods to estimate population size, they provide a 'reliability score', which '*take into account the derivation of original estimates and any methods used for*

extrapolation. As a general rule, estimates with reliability score 1 are based on direct counts with a minimum of extrapolation, those with reliability score 2 have been arrived at through extrapolation from reliable figures (or with a small amount of uncertainty around the estimate), while those with reliability score 3 were based on assumptions and opinion in place of actual fieldwork.'. For full details on the population estimates, see Musgrove et al.[2].

We filtered these data — extracted from Appendix 1 in Musgrove et al.[2] — by only including population estimates which: (1) were for Great Britain; (2) were provided for individuals or pairs; and (3) had a reliability of '1' or '2' (see above). We multiplied pairs by 2, to represent the estimated population size. If only a population range was given, we used the midpoint of the range as the population estimate.

BirdLife International population estimates. We extracted population estimates for species from BirdLife International datazone: http://datazone.birdlife.org/home in October 2019. These data are compiled by global experts for each species, generally representing the best estimate for each species' global abundance, usually presented in standard bands of estimated ranges (e.g., 1-49, 50-249, 250-999, 1,000-2,499, 2,500-10,000). Sometimes modelled estimates are provided for a species, where available. Because we needed to use these data as training inputs, we only used species' estimates that had relatively small ranges (that were < 60,000 square miles), helping the computation restrictions of collating eBird data through spatial queries. By incorporating these BirdLife population estimates, we broadened our training pool of species to

encompass more rare species as well. Where a standard band estimate was provided, we used the midpoint as the species abundance estimate in our modelling.

Estimating relative abundance from eBird data. Initially, we extracted three different measures of relative abundance from the eBird dataset for each of the geopolitical boundaries (or range size for BirdLife international estimates) which had associated data on population abundances from an external source (see SI Methods). This approach ensured that the spatial scale of the aggregated eBird data matched those of the abundance estimates provided by external sources, and this spatial scale was not uniform across species and regions. The three measures of relative abundance we assessed were: (1) modelled abundance at a given effort of time and distance; (2) a mean abundance across all checklists, including zeros for checklists on which a species was not found; and (3) the total abundance summed divided by the total time spent across all eBird checklists. For each measure, we used the reported number of individuals for a species on an eBird checklist, excluding an observation if it did not provide an estimate of abundance. Each variable was calculated by month, for each geo-political boundary, and all eBird checklists within that stratification were used for the calculations (i.e., we used all complete eBird checklists, as outlined above, regardless of whether they recorded a species of interest). This process was repeated for each of the species for which we had initial training data (N=724).

To model relative abundance, we used a Generalized Linear Model with a binomial logit link where the number of observations was the total number of eBird checklists in that

geo-political region. The response variable was binomial presence/absence, and the predictor variables were distance travelled during the eBird checklist and the duration of the eBird checklist — two ways to account for the differential effort among eBird sampling. We then used this fitted model and predicted the estimated abundance for a given 60-minute eBird checklist which travelled 1 km in distance. Initial exploration of the three measures of relative abundance revealed relatively strong collinear relationships, after removing the small percentage of models that did not converge and that had excessive standard errors. After subsetting the data to the months which resembled breeding (i.e., May, June, July, and August) or wintering for winter estimates in Great Britain, and taking the mean of these variables across any multiple Bird Conservation Regions that existed within a state, we found strong collinearity among relative abundance estimates from eBird (SI Appendix, Fig. S7). And because fitting GLMs on many different datasets would be redundant, we used the simplest measure of abundance: the mean abundance across all checklists (#2 in the list above).

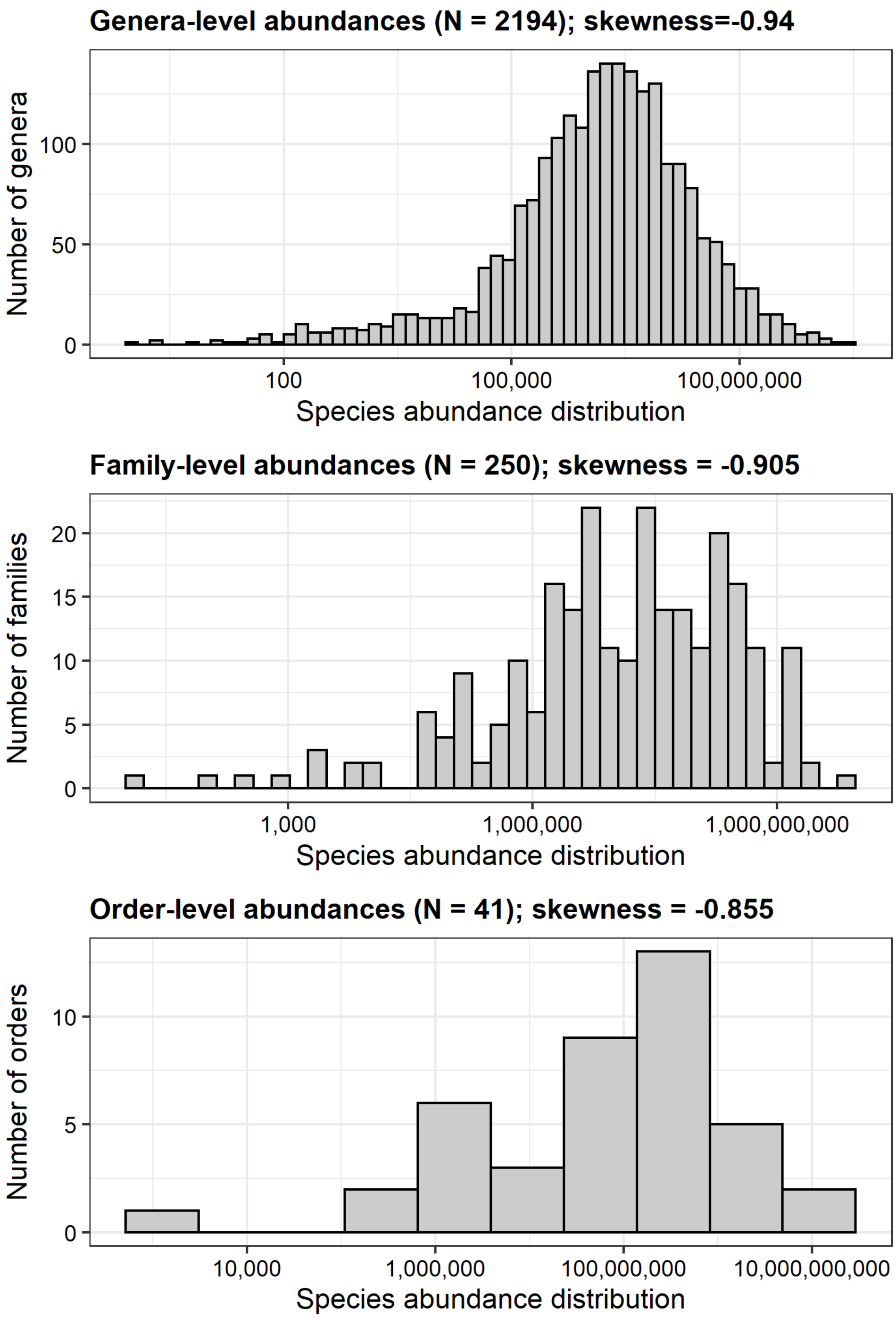

Figure S1. The abundance distributions at the genus, family, and order levels, with their associated skewness. Abundances were calculated by adding the species-specific abundance estimates for every species belonging to each respective genus, family, and order.

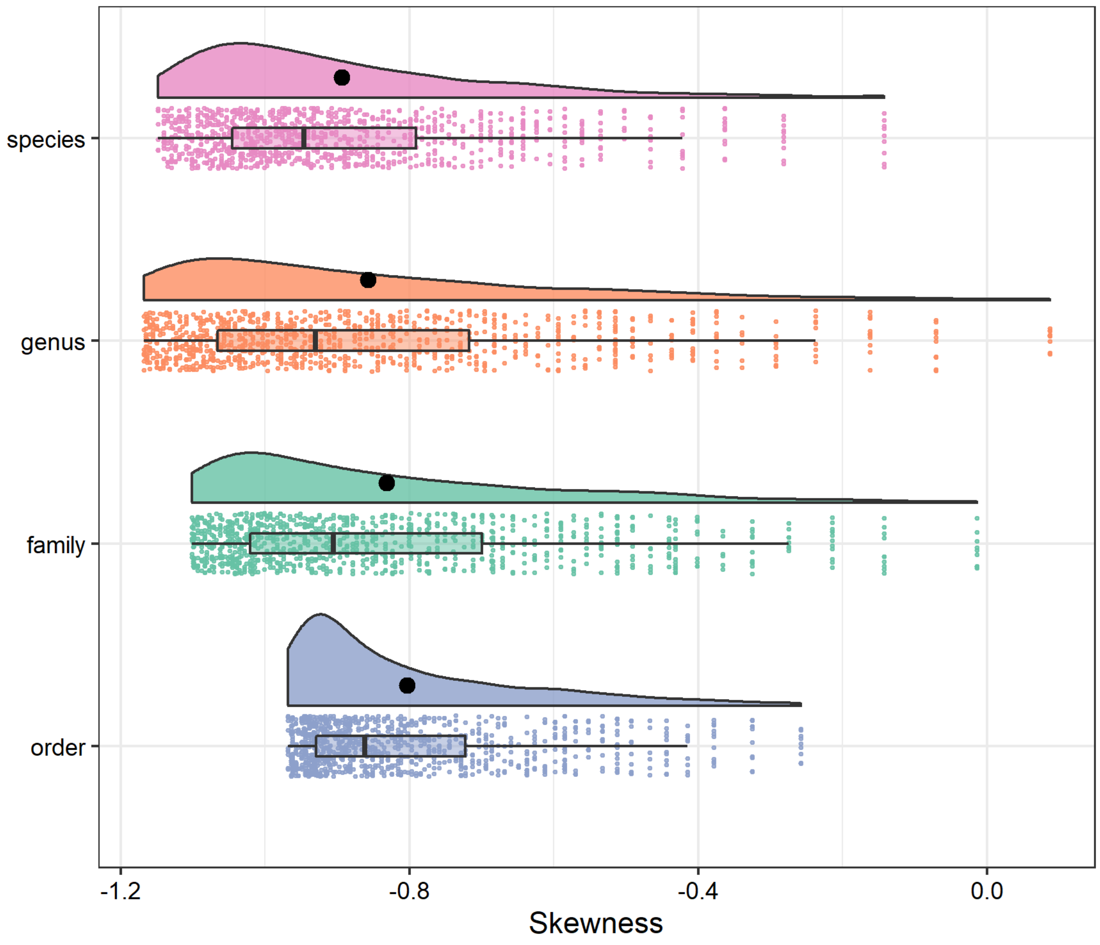

Figure S2. The results of our resampling approach showing the strong signal in the log left-skewed abundance distributions from species to order taxonomic levels. Resampling was done by randomly sampling quantiles from 0.1 to 0.99 and then calculating the abundance for every species using this quantile (as opposed to the median which is used throughout). The black dot represents the mean for each taxonomic group.

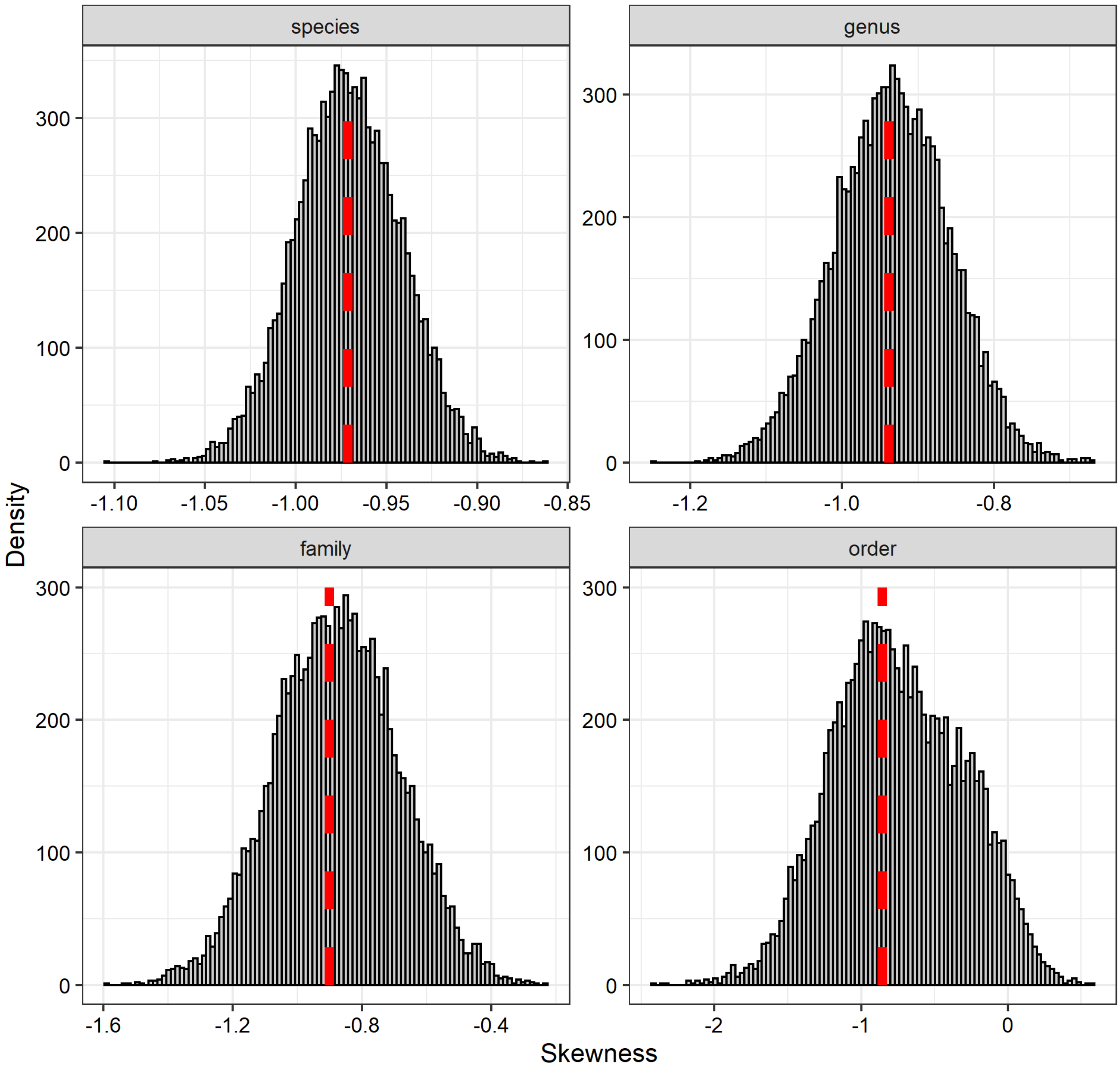

Figure S3. Results of a bootstrapping analysis (N=10,000) showing the consistent pattern in log-left skewness at species, genus, family, and order taxonomic levels. The red line represents the mean skewness family for the respective taxonomic level. These results confirm that of our resampling approach presented in Figure S2.

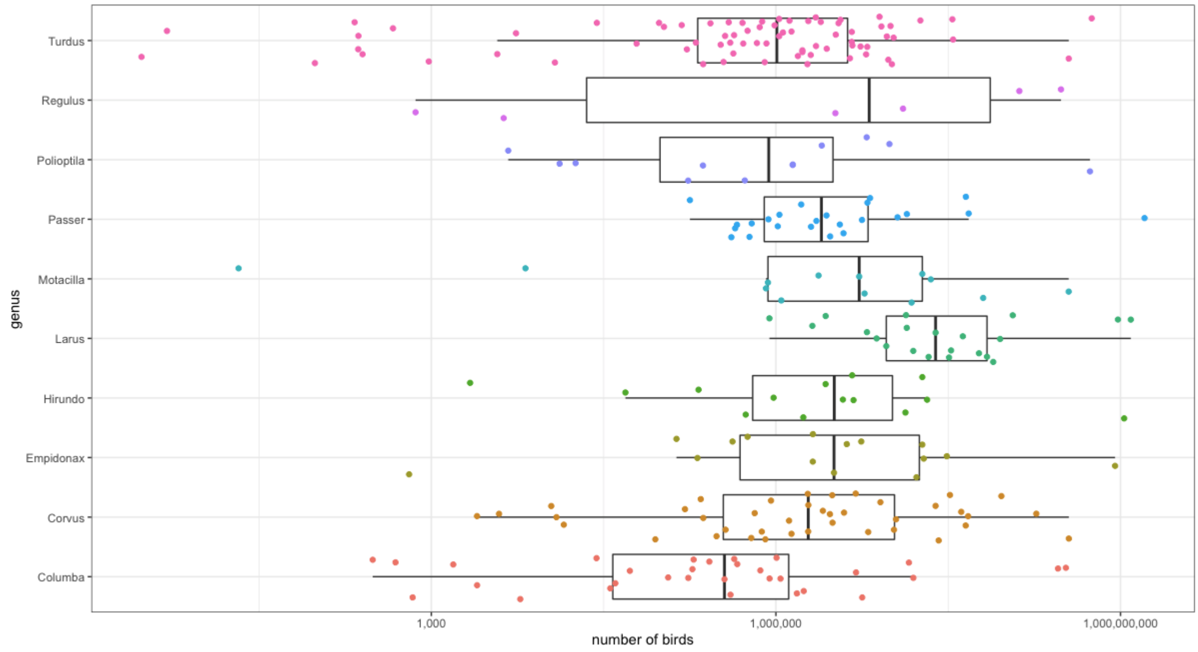

Figure S4. The abundance of species within genera with both a top-10 most abundant species and a species richness of greater than 5, illustrating that even abundant clades can have exceptionally rare species (e.g., Turdus).

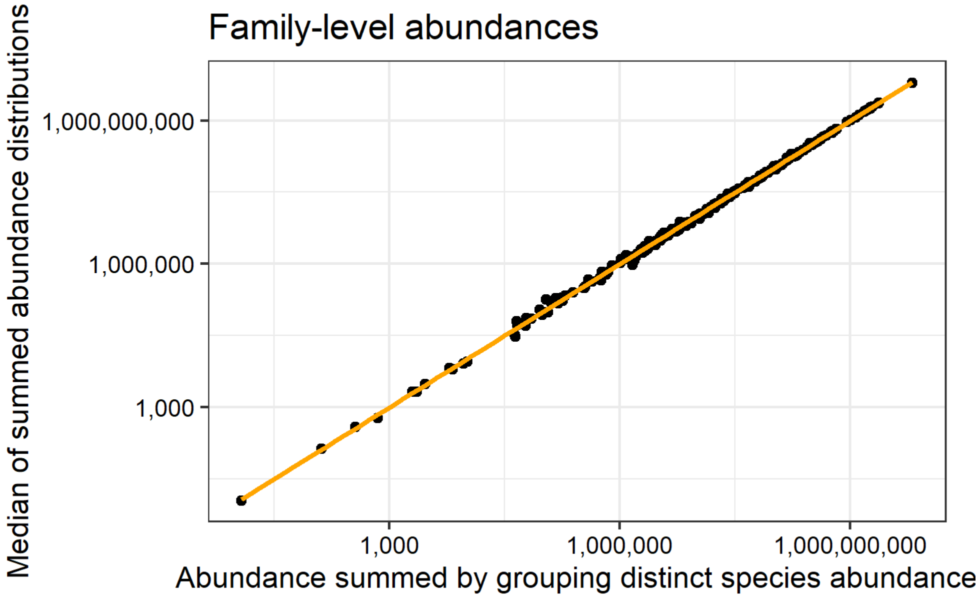

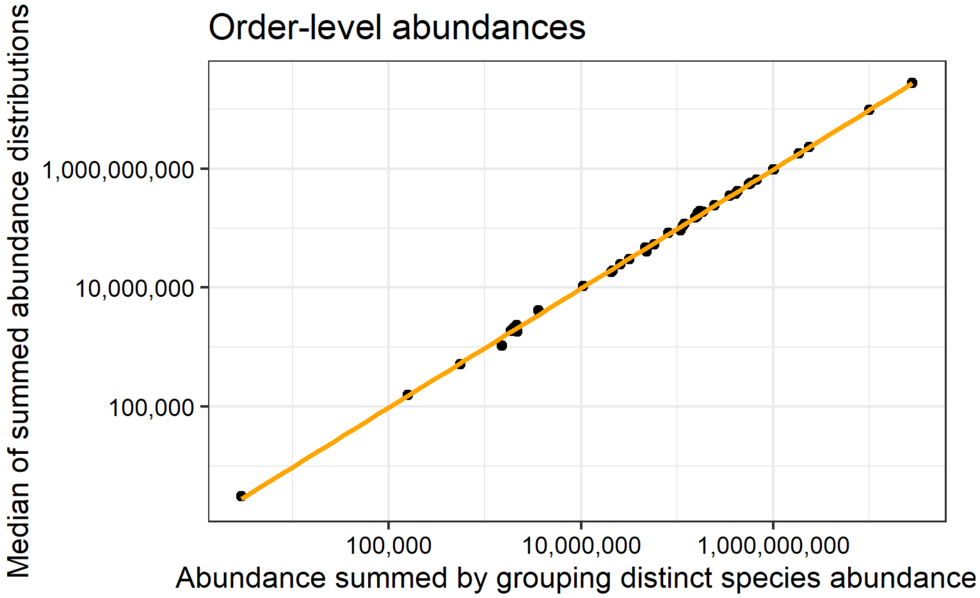

Figure S5. Scatterplot showing the relationship between family-level (top) and order-level (bottom) abundance measures calculated in two different manners. First, on the x-axis, abundance is calculated by using the species-specific abundance estimate (i.e., the median of a species' abundance distribution), and second, on the y-axis, abundance is calculated by summing the abundance distributions for each species within a category and taking the median of the summed abundance distribution.

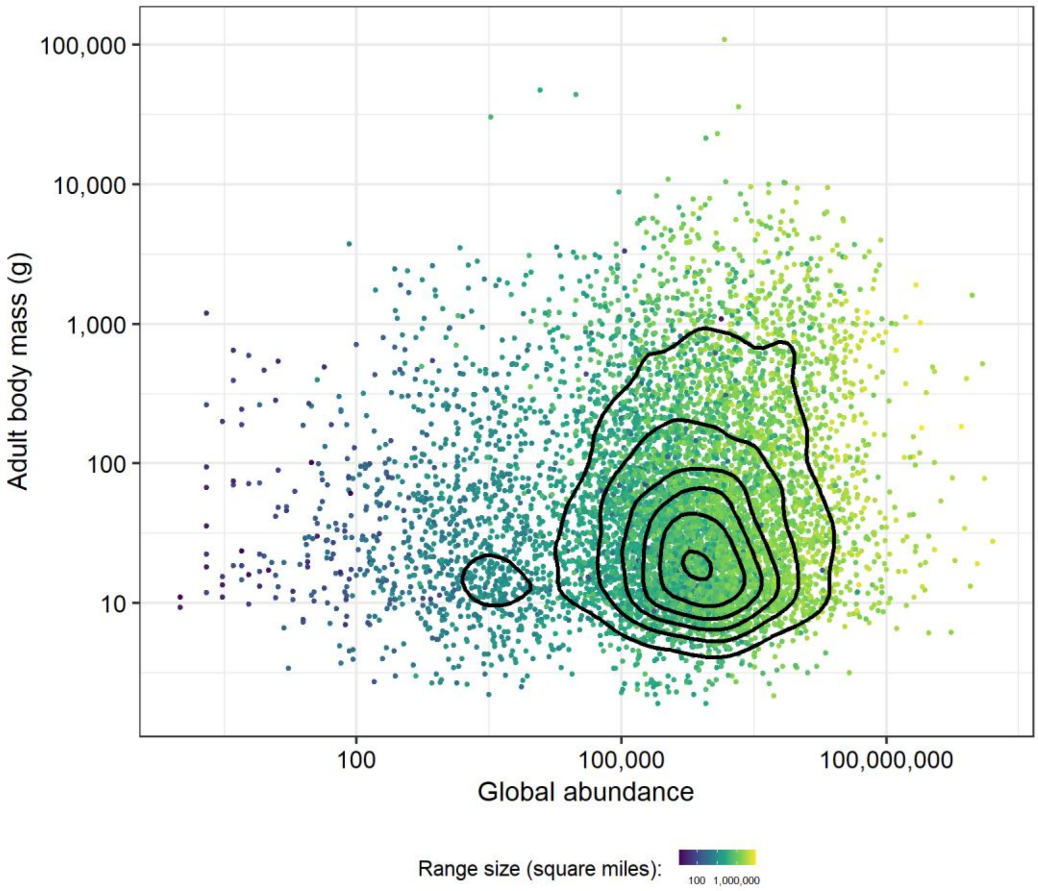

Figure S6. The relationship between global abundance, body mass, and range size, showing a slightly positive relationship. The black line represents a 2D density plot of the data. Data are shown for 7,717 species.

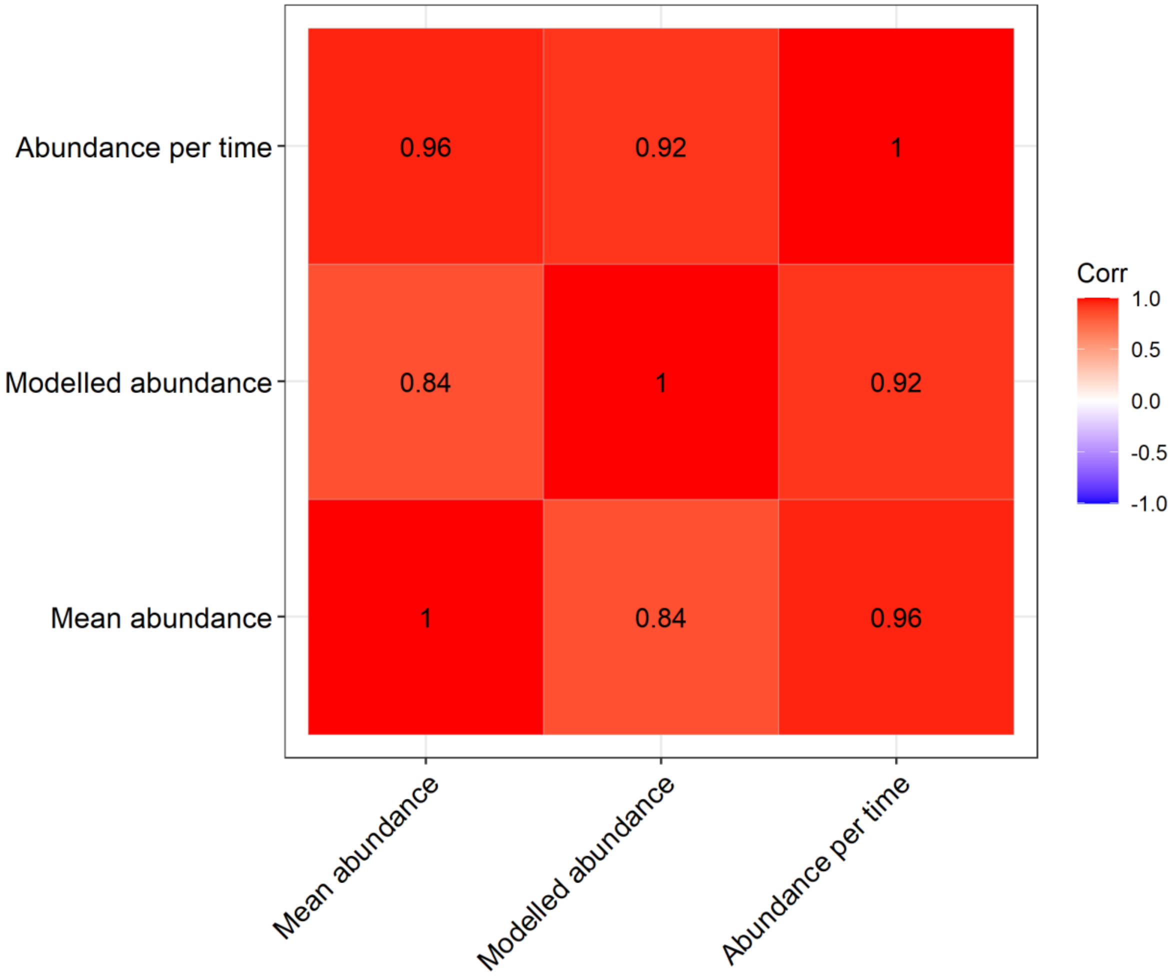

Figure S7. The relationship between our three potential measures of relative abundance from eBird, taken as the mean for each species (N=724). For each measure, the average is taken over the time corresponding to the abundance measures (for Birdlife species, the relative abundance measures are averaged over the entire year; for Partners in Flight species, the relative abundance estimates are averaged over the breeding season; for British estimates the relative abundance was averaged over either the breeding season or the wintering season). For the modelled estimates, models which did not converge or were below the 0.1 and above the 0.99 quantile of model fit were excluded from comparisons. Because of the strong collinearity among all three measures, we used the mean abundance across all checklists as our relative abundance measure throughout our analysis.

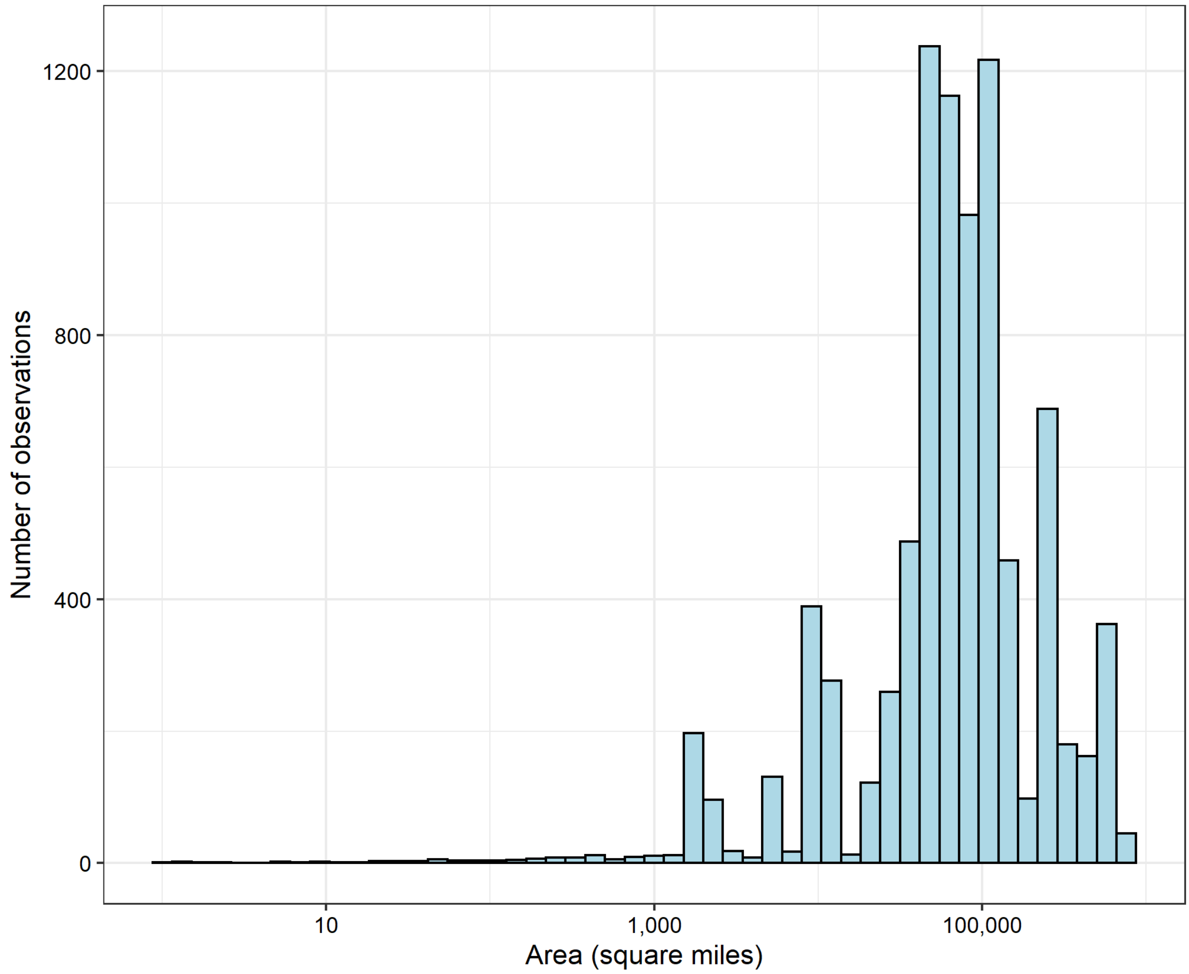

Figure S8. The distribution of areas used to calculate relative abundance for our 724 initial training species.

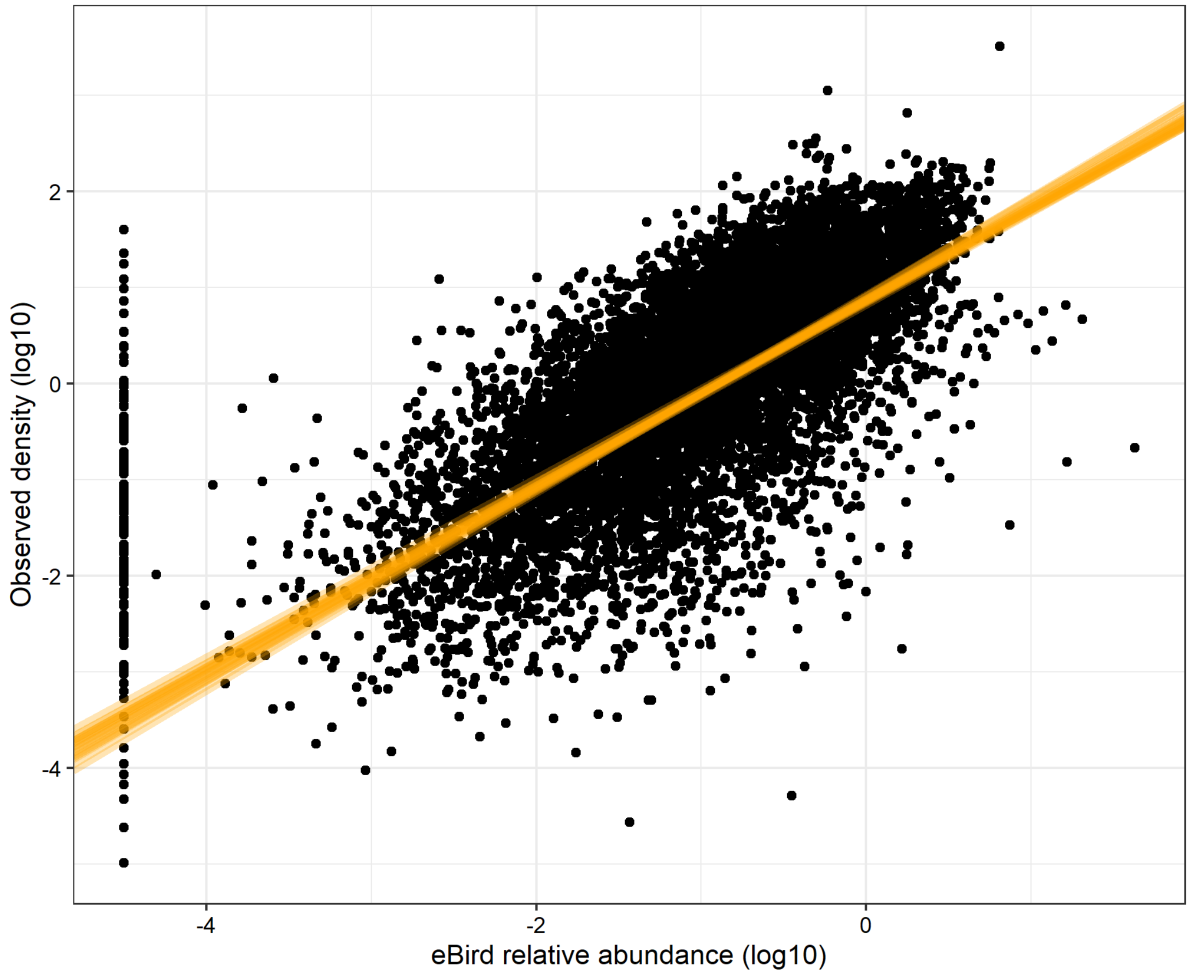

Figure S9. The relationship between observed density and eBird relative abundance for 724 training species used in our brms modelling process (see methods). The orange lines represent 20 posterior draws from the model fit representing the fixed effects. Note that for species with 0 relative abundance, we set these to the minimum log10 relative abundance in our dataset, at ~ -4.5.

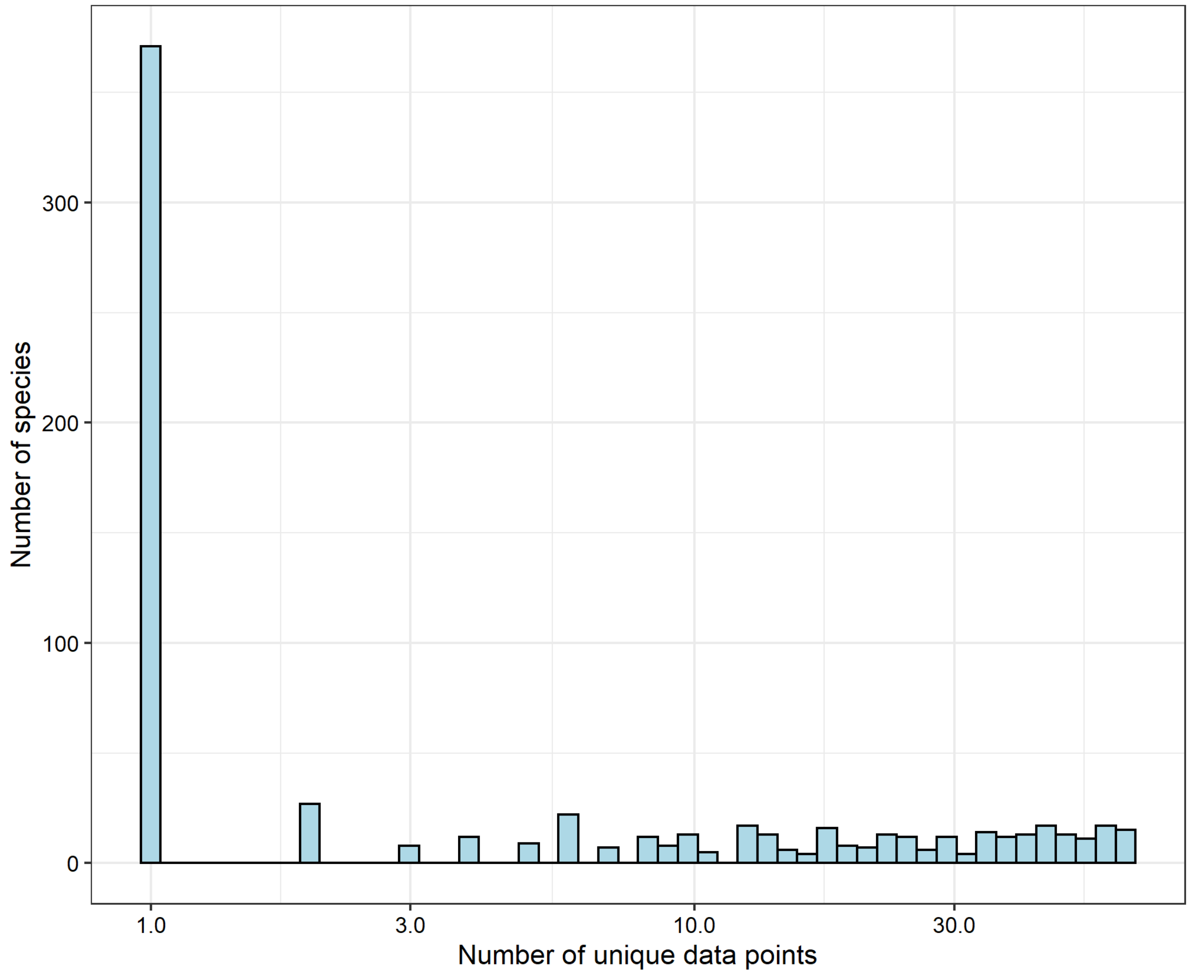

Figure S10. A histogram showing the number of data points, per species, used in the analysis. Most species had 1 observation in the brms model (see Figure S8), but some species (i.e., those from the United States) had relatively many data points in the model (see Figure S11).

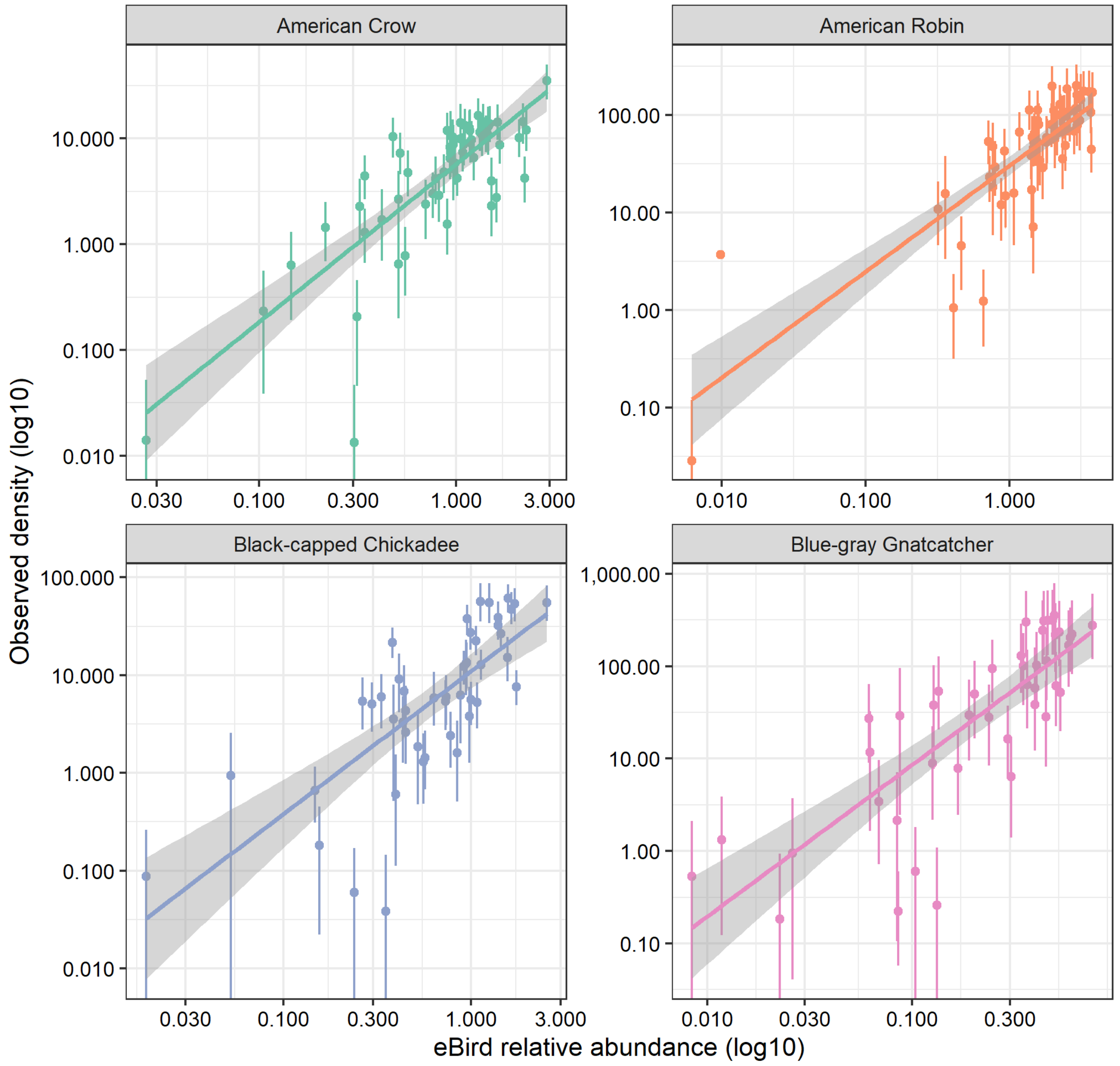

Figure S11. Four example species, showing the relationship between eBird relative abundance (i.e., the mean abundance across all checklists) and the observed density (i.e., extracted from the Partners in Flight database). Both variables are log10-transformed.

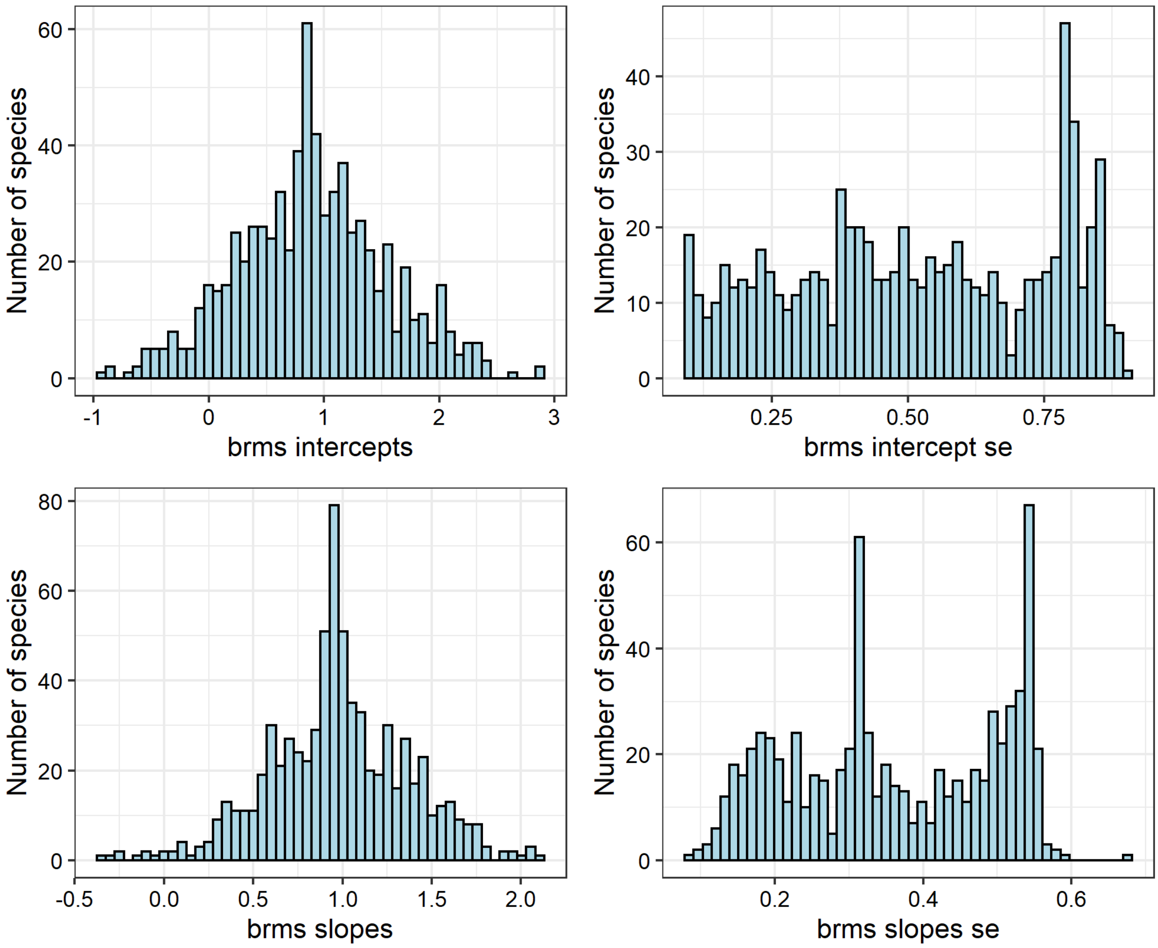

Figure S12. The results of the brms model for intercepts, and slopes, extracted for all 724 species used in our training dataset.

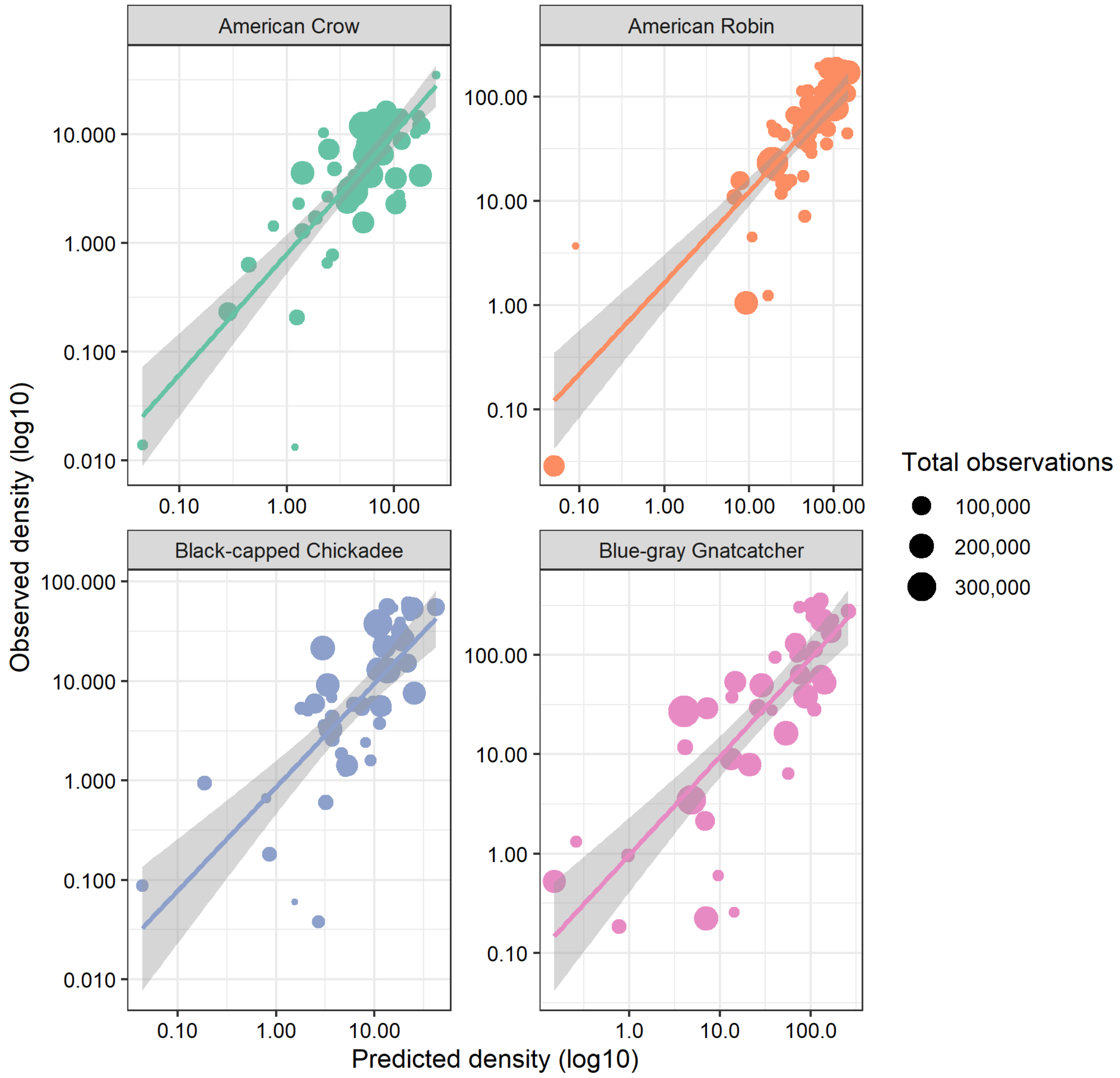

Figure S13. The four example species previously highlighted in Figure S10, showing the relationship between predicted density and observed density, and the number of observations for each independent data point used in the brms modelling. We did this for every species, and calculated the total abundance, and these results are shown in Figure S14.

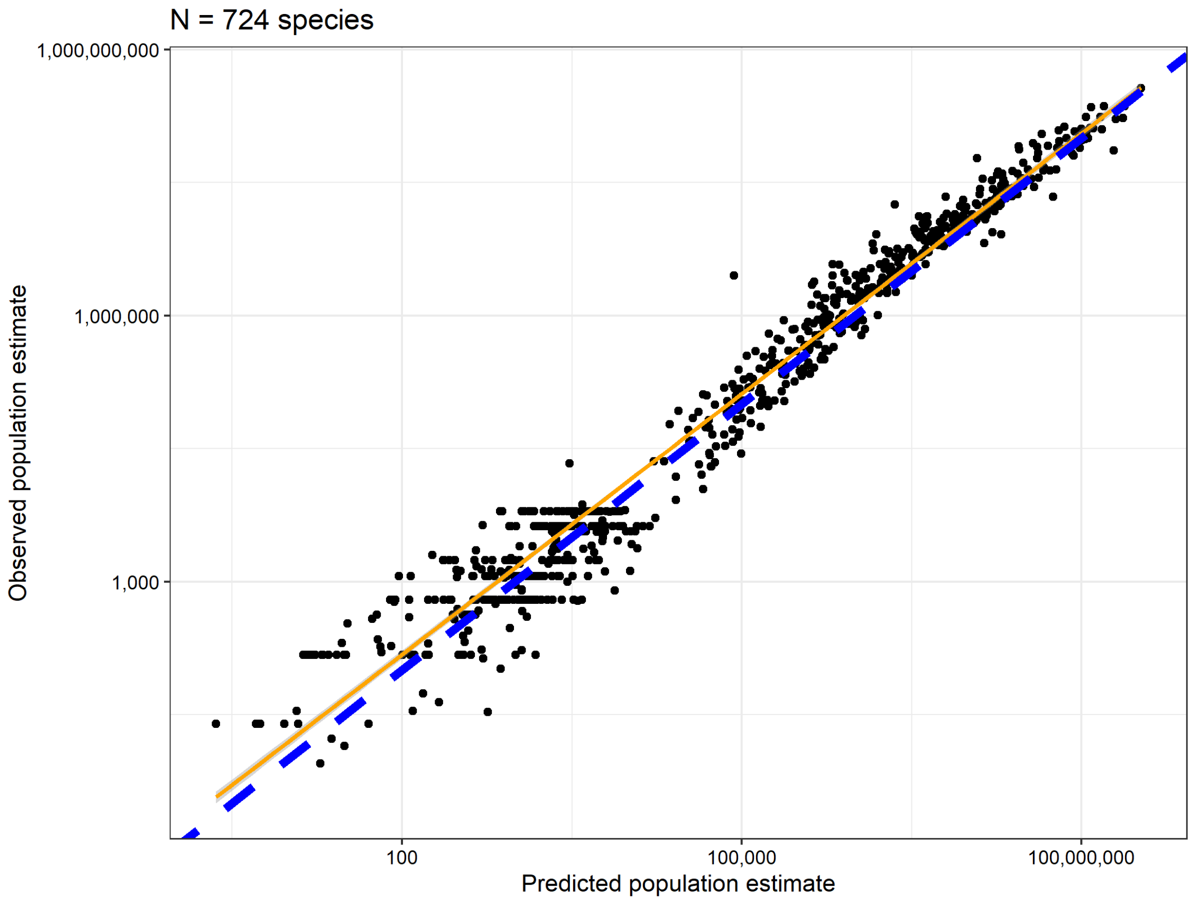

Figure S14. The relationship between predicted population estimates, using our intercept and slope extracted from the brms model (see methods), and the observed population estimate. The orange line represents a linear model fit, whereas the blue line represents a line with slope=1 and intercept = 0. Overall, we found that our brms modelling strongly predicted the observed population estimate (R^2=0.88).

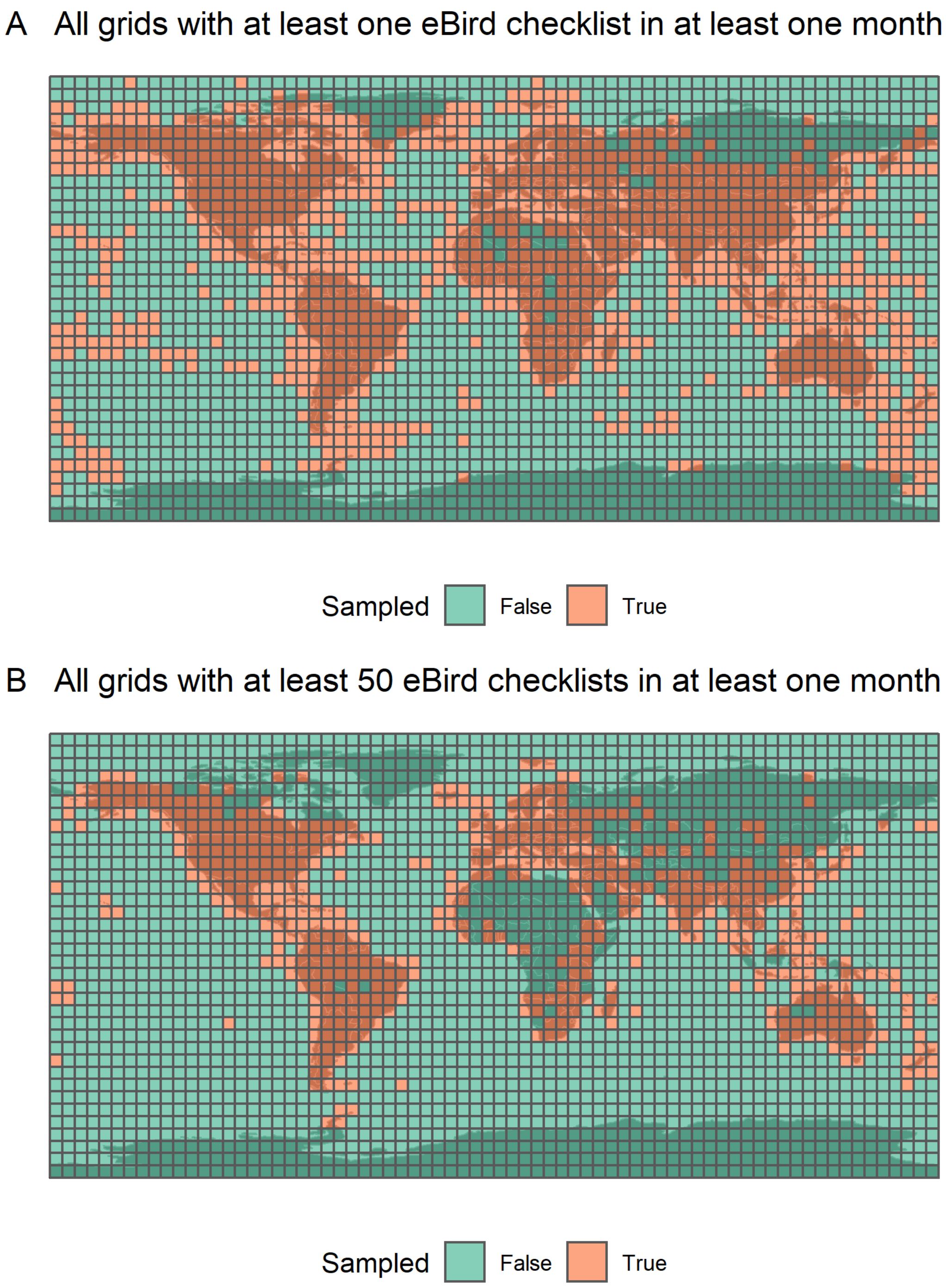

Figure S15. A) The 5-degree grid cells used in our analysis and all grids which have at least one eBird checklist, and B) the 5-degree grid cells used in our analysis, showing grids which have at least 50 eBird checklists within at least one month. Only grids in B) were used in analysis to calculate relative abundance.

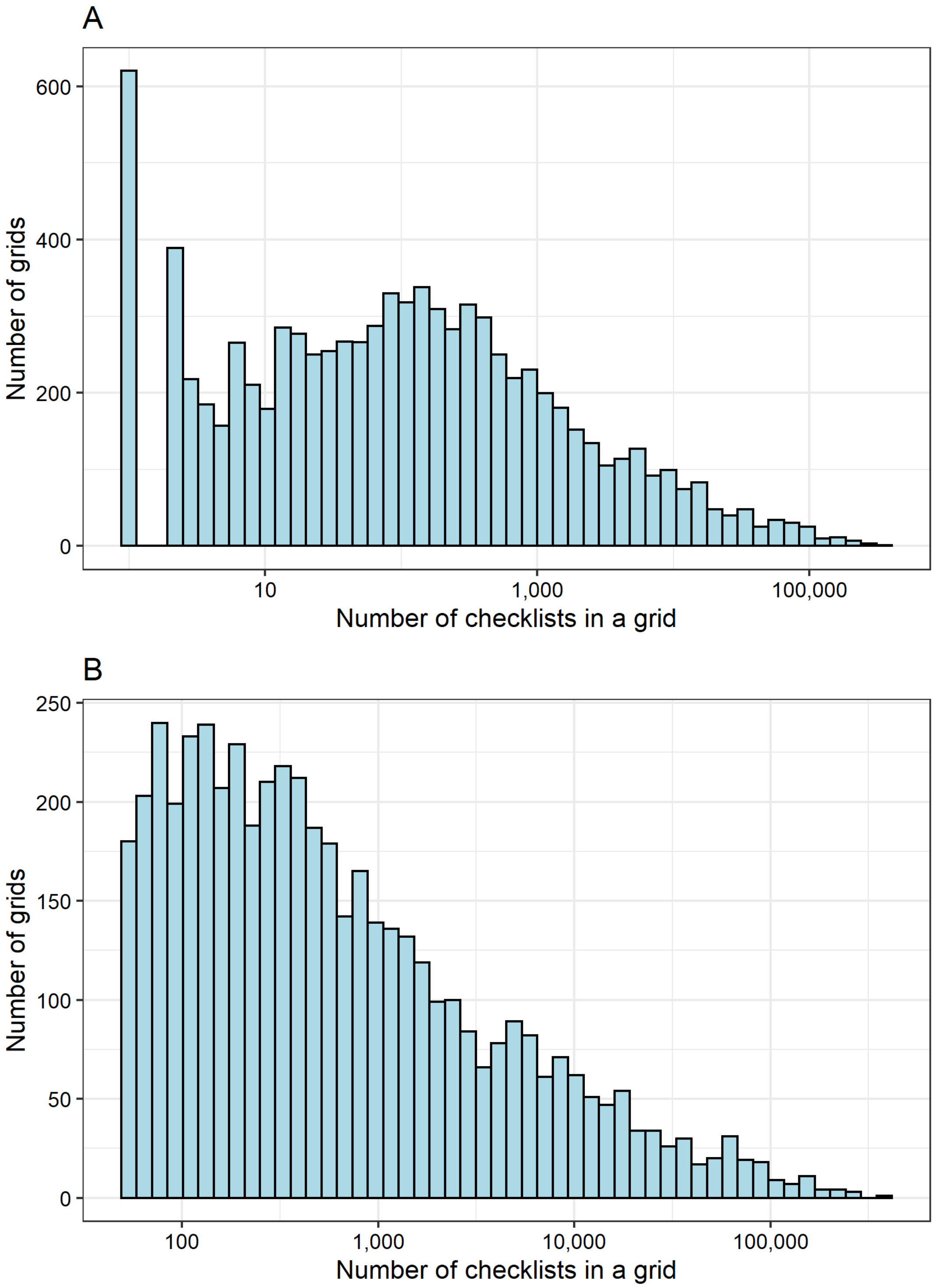

Figure S16. A) The number of eBird checklists per grid, including any grid that had at least one eBird checklist, and B) the number of eBird checklists per grid, for all grids with at least 50 eBird checklists in at least one month (Figure S14), which were used in analyses.

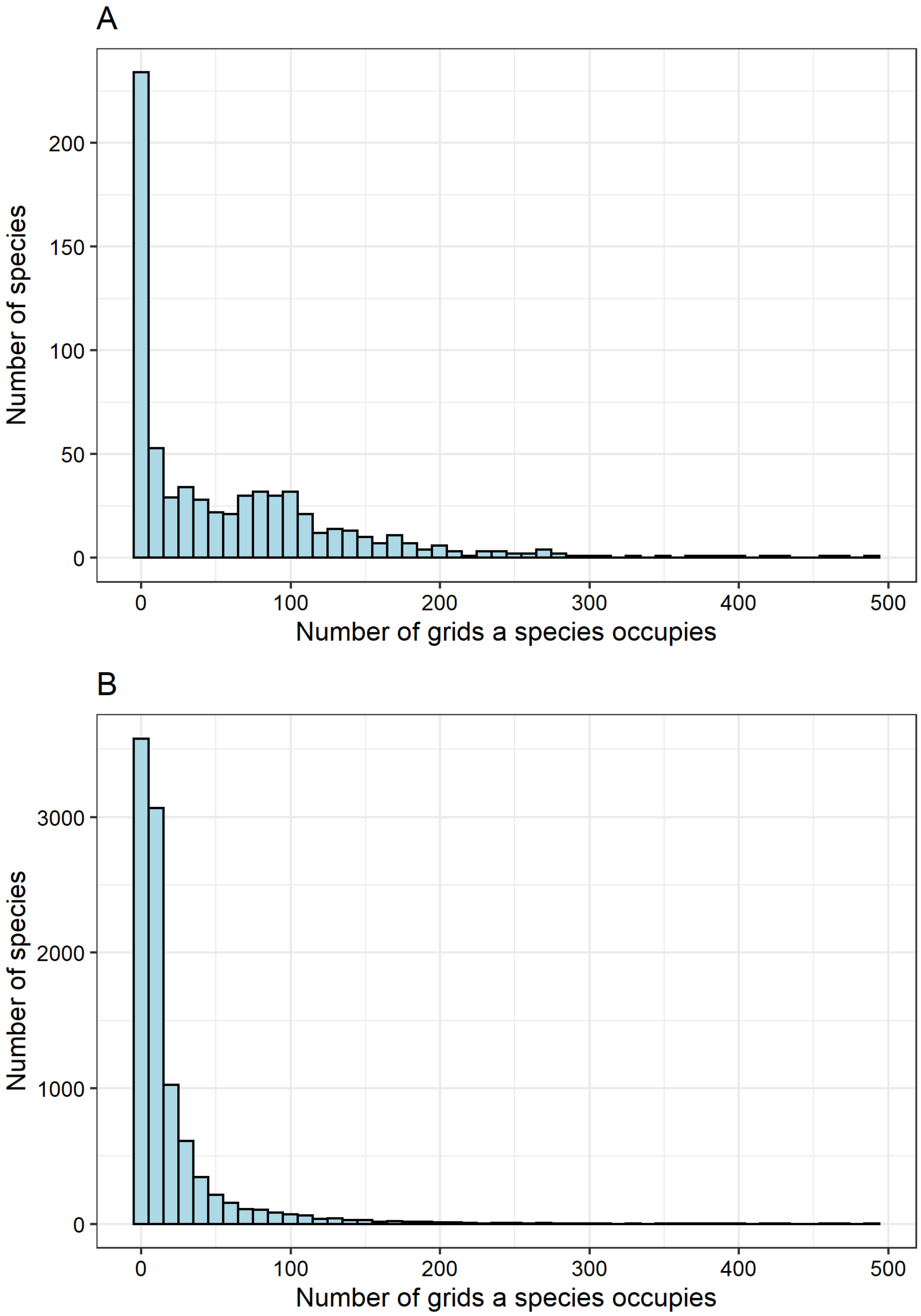

Figure S17. The number of 5x5 degree grids that our training species (A) were found in and all species included in our analysis were found in (B).

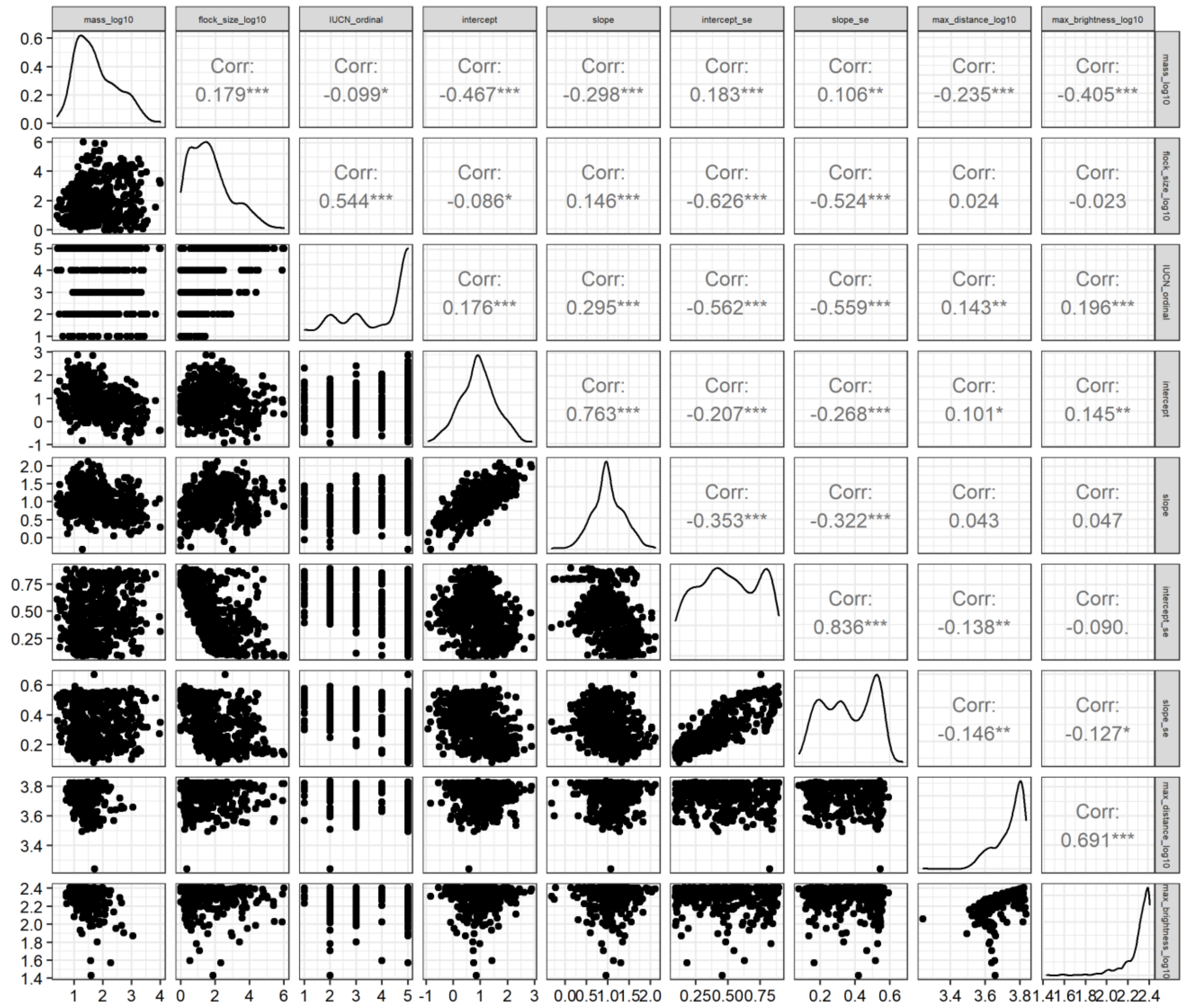

Figure S18. The relationships for 684 species, between intercept and slope of the brms model and life history traits used in the imputation. From left to right across the top, the following categories are represented: mass_log10, flock_size_log10, IUCN_ordinal, intercept, slope, intercept_se, slope_se, max_distance_log10, max_brightness_log10. And from top to bottom across the right hand side the following are represented: mass_log10, flock_size_log10, IUCN_ordinal, intercept, slope, intercept_se, slope_se, max_distance_log10, max_brightness_log10. The figure was made using ggpairs from the GGally package in R.

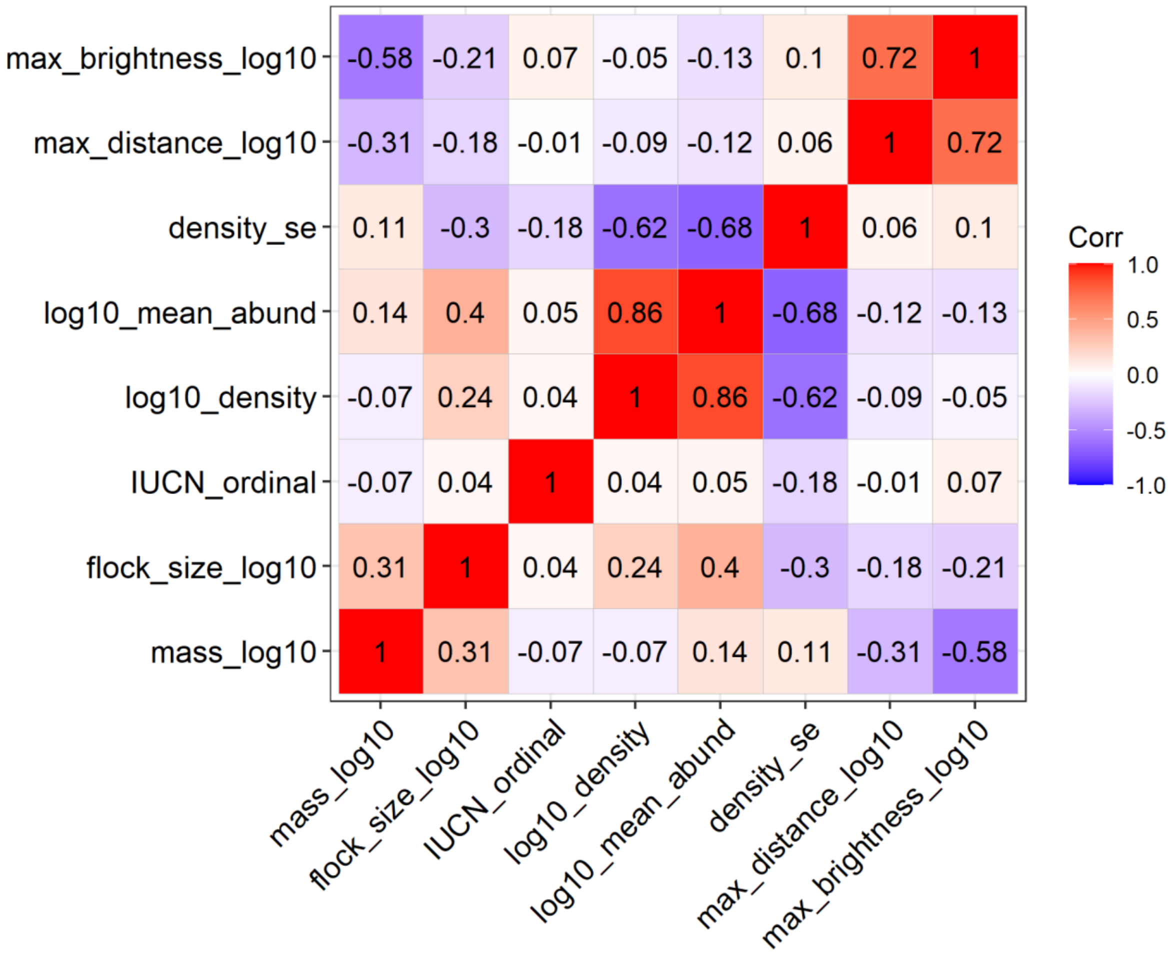

Figure S19. The pairwise relationships for the training data used in the imputation. Pairwise complete observations are shown. IUCN is an ordinal variable.

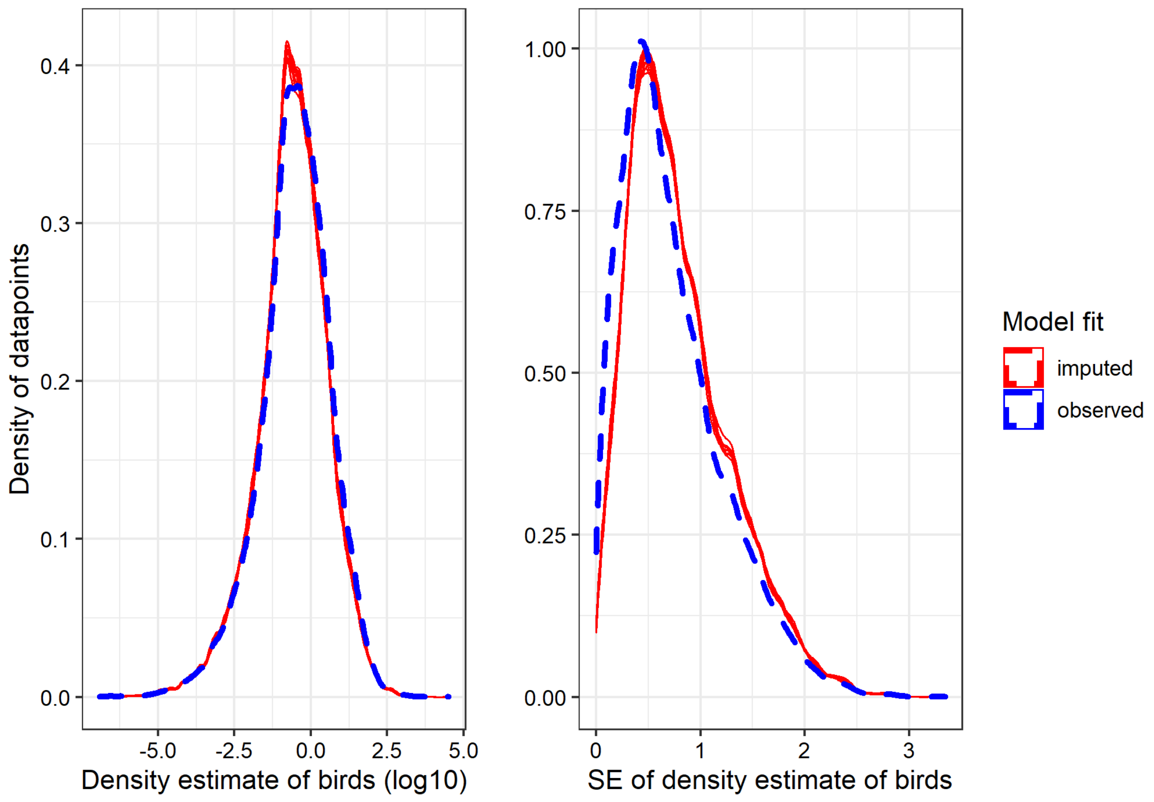

Figure S20. Density histograms showing the density of observations for both the density estimate of birds (left) and the standard error of the density estimate of birds (right) for both the observed values used in imputation (blue dashed line) and imputed values for ten random imputations (red lines). The similarity in observed and imputed density histograms indicates that the imputation produced plausible values statistically.

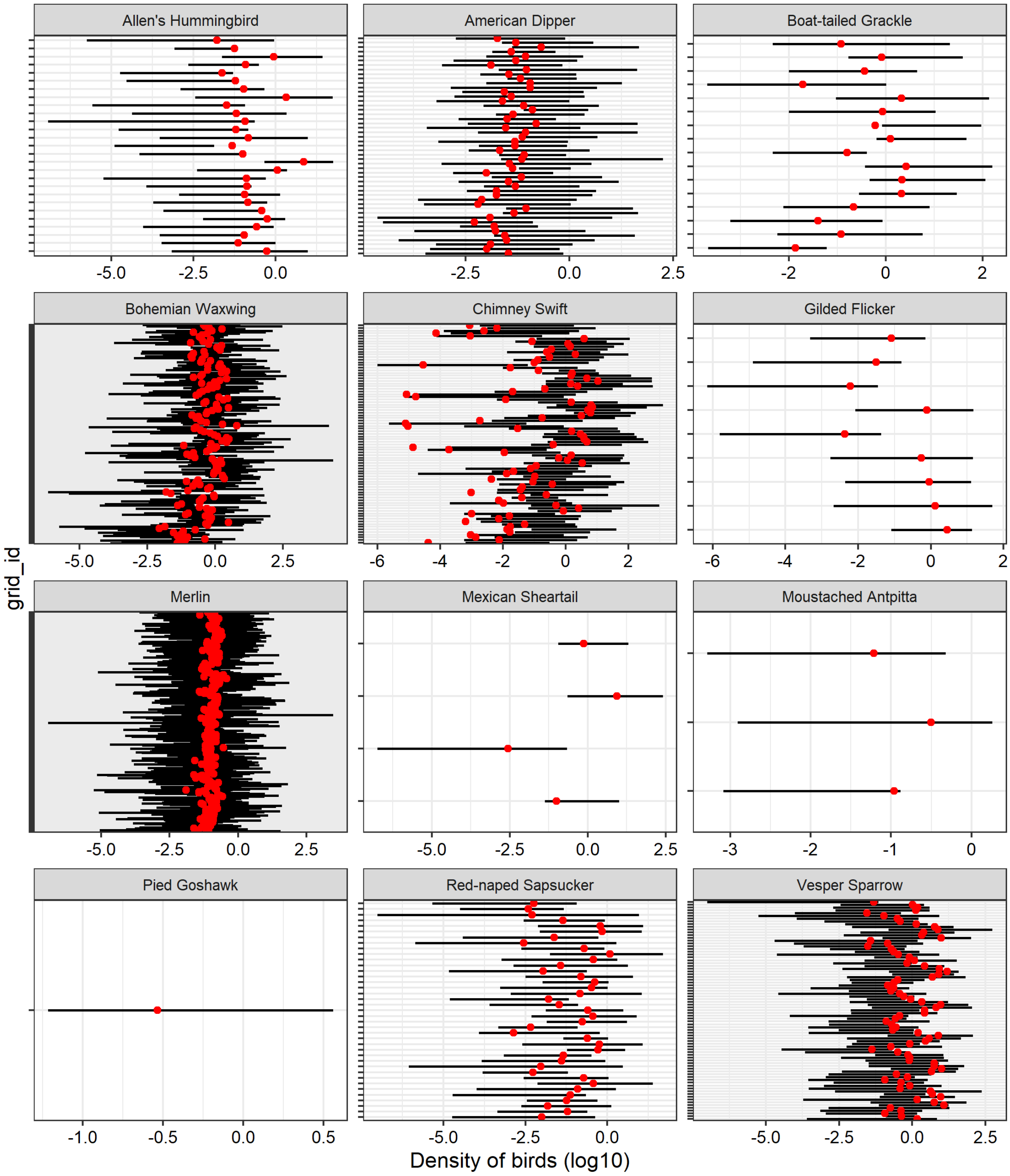

Figure S21. Twelve example species showing their observed density estimate per grid cell (red dot) and the range of imputed density estimates across 10 imputations (black line).

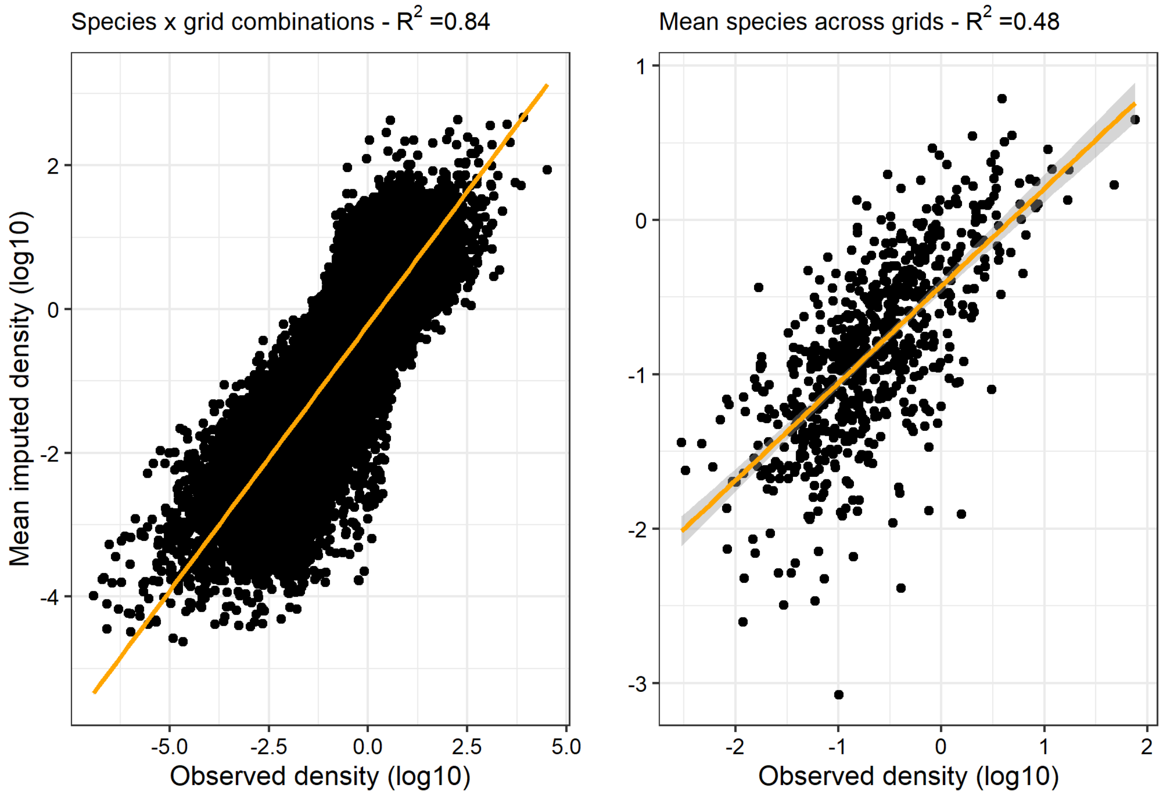

Figure S22. The relationship between observed density and the mean imputed density among ten imputations for each unique species x grid combination (left panel) and for each species' mean density across all grids (right panel).

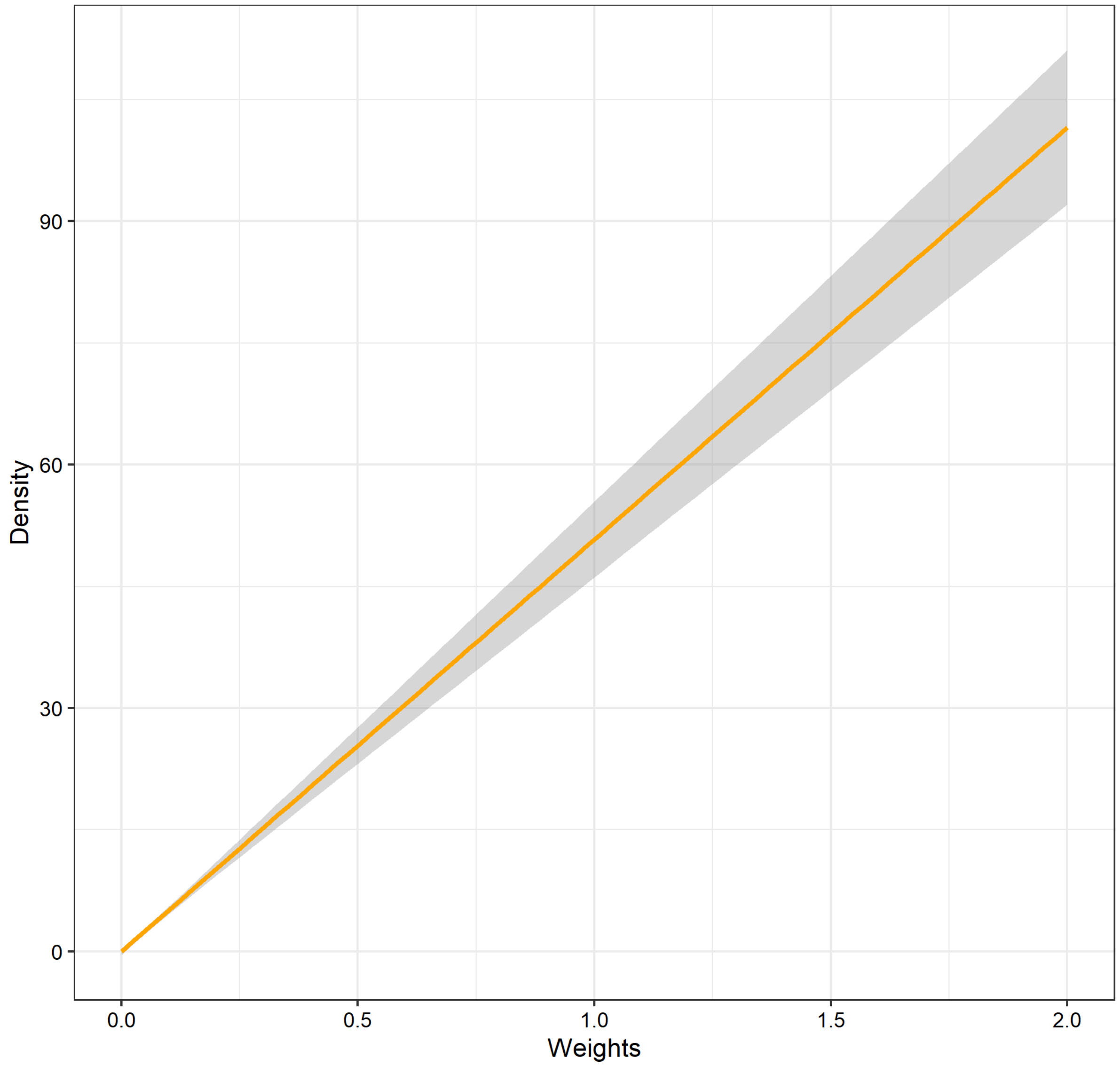

Figure S23. The relationship between density in a grid cell and the 'weights' used to calculate the weighted density. Weights were defined as the total number of checklists a species was found on divided by the total number of checklists in a grid cell. For a species found in more than one grid cell, the overall density of that species (Figure S17) was calculated by taking the weighted mean, providing more confidence to grid cells where the species is most frequently observed and grid cells that have more checklists in them.

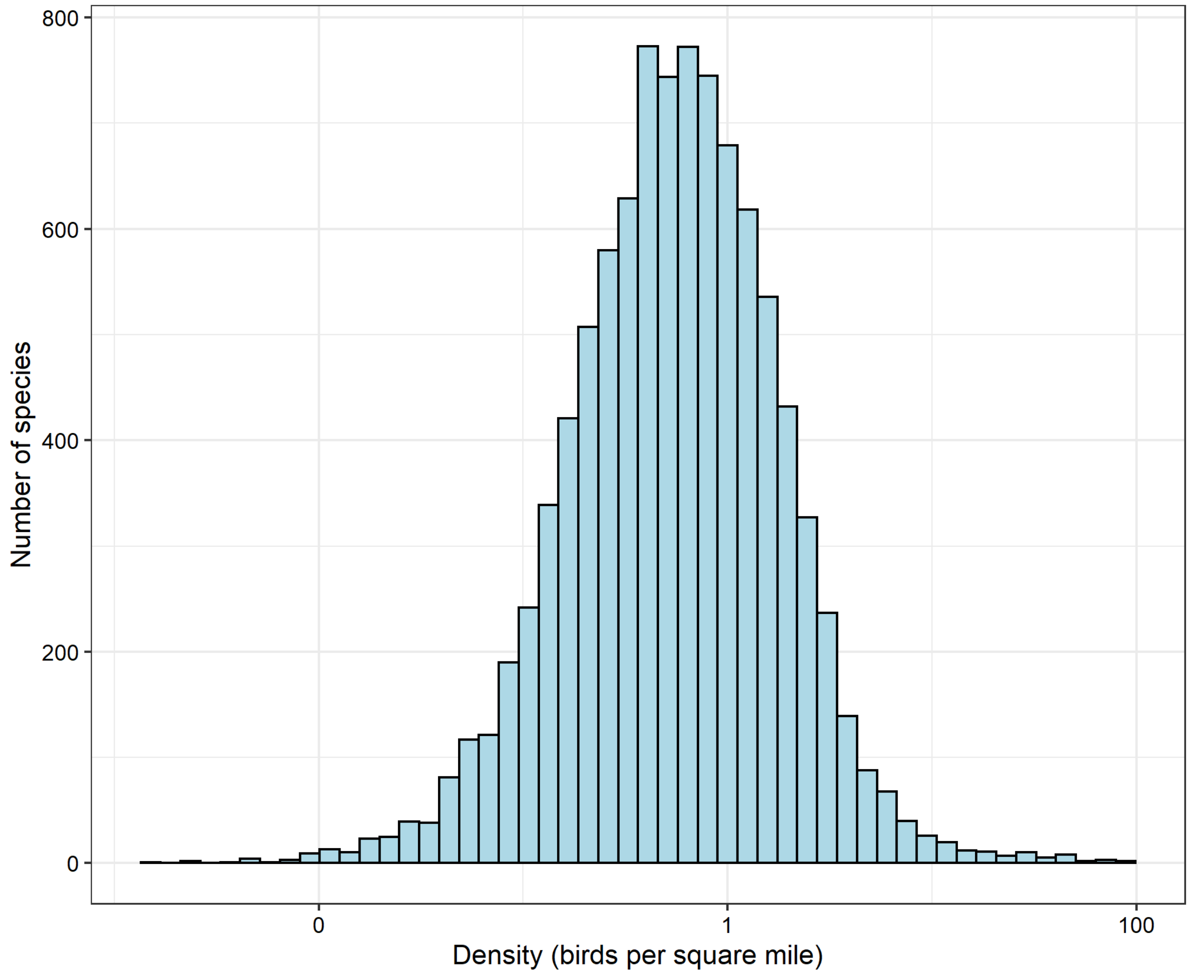

Figure S24. The distribution of weighted densities for 9,700 species (on a log10 scale). The weighted density was calculated by weighting the density estimates by the number of checklists a species was on divided by the number of total checklists in a grid cell (see Figure S23).

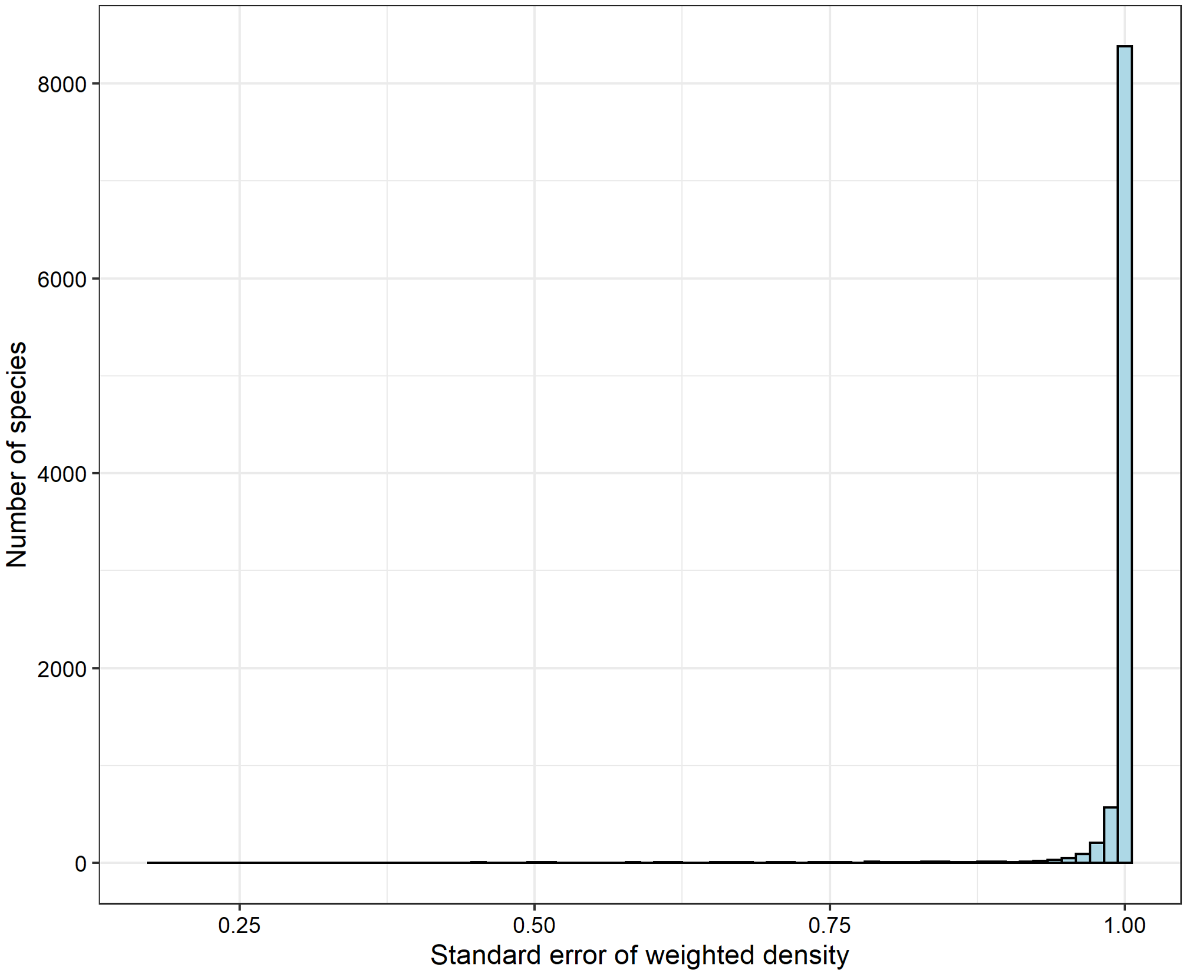

Figure S25. The distribution of standard errors of our density estimates, where we set a ridge prior of 1, equating to a maximum of 91 individuals more per square mile.

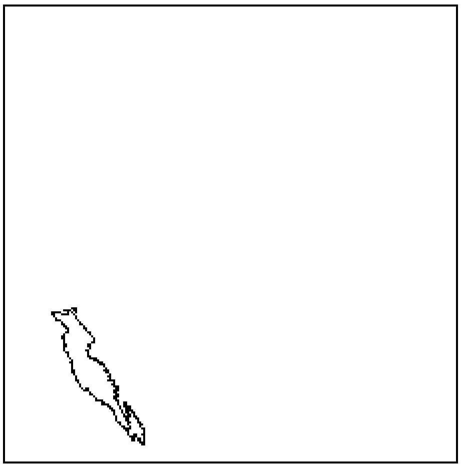

Figure S26. The range of the Red-bellied Myzomela, an endemic species to Malaita, Solomon Islands, and the corresponding grid cell that species belongs to. In this instance, the species' range was clipped from the grid range to the species' range size.

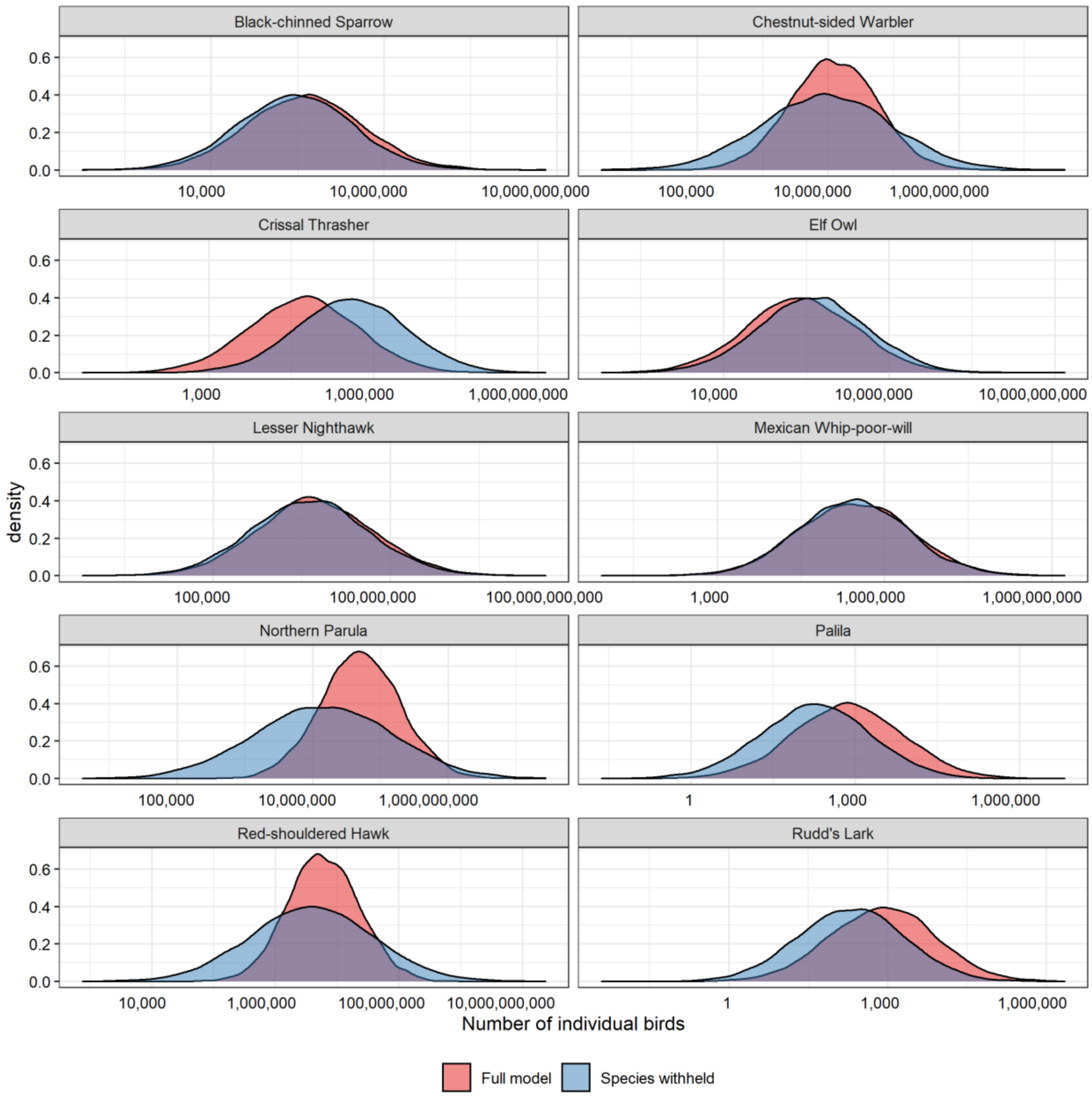

Figure S27. Ten randomly chosen example species and their estimates of abundance distributions from our analysis for the full model when all 684 species were included as training species (red) and when that species was withheld as a training species.

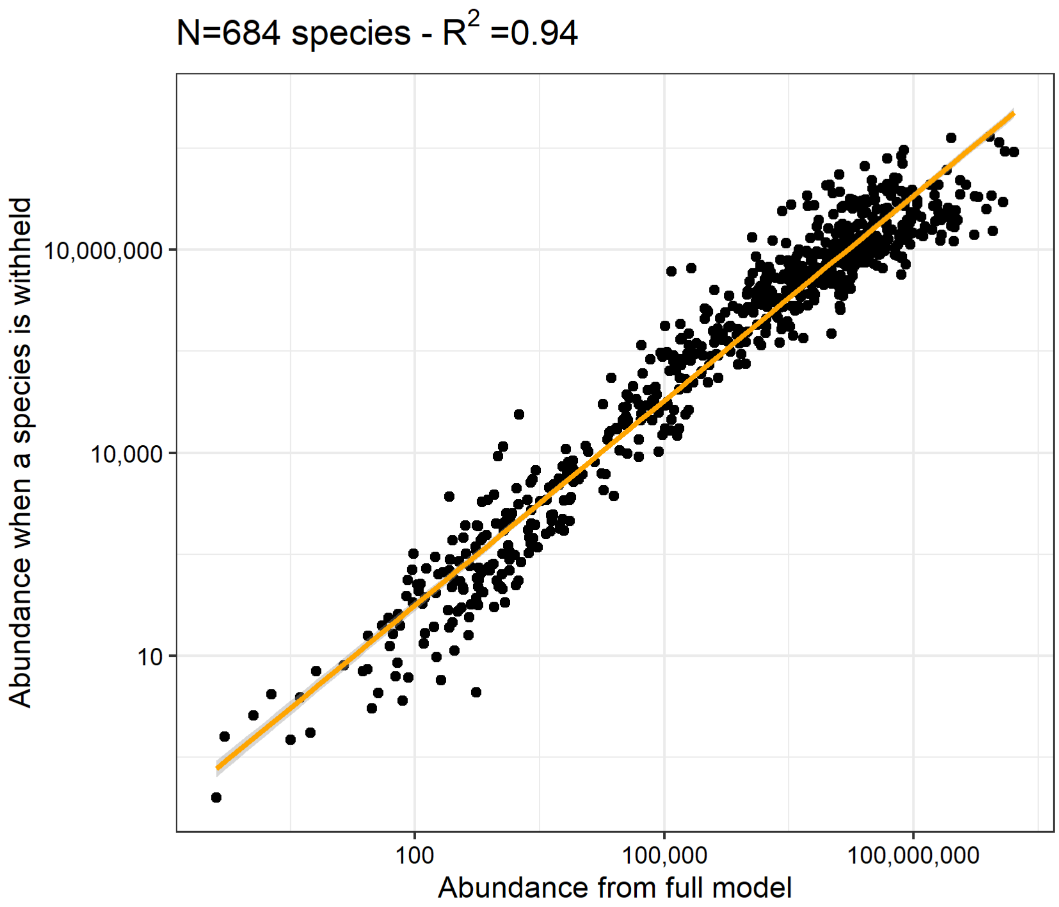

Figure S28. The relationship between predicted global abundance from our full model workflow (x-axis) and predicted global abundance when a species was not included as a training species (y-axis), for 684 species.

Dataset S1 (separate file). Each of the 9,700 species included in our analysis, and their abundance estimate, with 95% upper and lower confidence intervals. We also note whether or not a species was a training species and whether or not the range of each species was adjusted (see methods for details).

SI References

1. Will, T. *et al.* Handbook to the partners in flight population estimates database, version 3.0. *PIF Technical Series* (2019).

2. Musgrove, A. *et al.* Population estimates of birds in Great Britain and the United Kingdom. *British Birds* **106**, 64–100 (2013).

3. Rich, T. North American landbird conservation plan (2004).

4. Rosenberg, K. V. & Blancher, P. J. Setting numerical population objectives for priority landbird species. In *In: Ralph, C. John; Rich, Terrell D., editors 2005. Bird Conservation Implementation and Integration in the Americas: Proceedings of the Third International Partners in Flight Conference. 2002 March 20-24; Asilomar, California, Volume 1 Gen. Tech. Rep. PSW-GTR-191. Albany, CA: US Dept. of Agriculture, Forest Service, Pacific Southwest Research Station: p. 57-67*, vol. 191 (2005).

5. Stanton, J., Blancher, P., Rosenberg, K., Panjabi, A. & Thogmartin, W. Estimating uncertainty of North American landbird population sizes. *Avian Conservation and Ecology* **14** (2019).

6. Brewer, R. Atlas of the breeding birds of Ontario, 2001–2005 (2009).

7. Sauer, J. R., Link, W. A., Fallon, J. E., Pardieck, K. L. & Ziolkowski Jr, D. J. The North American breeding bird survey 1966–2011: summary analysis and species accounts. *North American Fauna* **79**, 1–32 (2013).

8. Thogmartin, W. E. *et al.* A review of the population estimation approach of the north american landbird conservation plan. *The Auk* **123**, 892–904 (2006).

9. Thogmartin, W. E. Sensitivity analysis of North American bird population estimates. *Ecological Modelling* **221**, 173–177 (2010).

10. Matsuoka, S. M. *et al.* Using binomial distance-sampling models to estimate the effective detection radius of point-count surveys across boreal Canada. *The Auk* **129**, 268–282 (2012).

11. Rosenberg, K. V., Blancher, P. J., Stanton, J. C. & Panjabi, A. O. Use of North American breeding bird survey data in avian conservation assessments. *The Condor: Ornithological Applications* **119**, 594–606 (2017).
